# Keratin 20 deficiency phenocopies human UAB and drives bladder fibrosis via a mechanotransduction-TGF-β axis

**DOI:** 10.1016/j.isci.2026.115905

**Published:** 2026-04-27

**Authors:** Jun Jiang, Yongjia Bao, Tao Huang, Hanbo Zhang, Wensu Ma, Junwei He, Xianbin Duan, Chenxi Mo, Rui Guo, Jingjie Chen, Fang Yan, Jiehui Chen, Xing Liu, Yichen Huang, Fang Chen, Jiasheng Chen, Congcong Dong, Chunming Guo

**Affiliations:** 1 Yunnan Key Laboratory of Cell Metabolism and Disease, and Center for Life Sciences, School of Life Sciences, Yunnan University, Kunming, Yunnan 650500, China; 2 State Key Laboratory of Cell Biology, CAS Center for Excellence in Molecular Cell Science, Shanghai Institute of Biochemistry and Cell Biology, Chinese Academy of Sciences, 320 Yue Yang Road, Shanghai 200031, China; 3 Department of Urology, Children’s Hospital of Chongqing Medical University, 136 Zhongshan Road, Chongqing 400014, China; 4 Department of Urology, Shanghai Children’s Hospital, School of Medicine, Shanghai Jiao Tong University, Shanghai 200062, China; 5 Guangdong Hybribio Biotech CO., Ltd., Chaozhou, Guangdong 521000, China

**Keywords:** Mechanobiology, Fibrosis, Molecular biology

## Abstract

Lower urinary tract symptoms (LUTS) represent a spectrum of intractable and progressive disorders. A common pathological feature of LUTS is bladder fibrosis. Here, we established an original mouse model of urinary retention and underactive bladder by ablating urothelial keratin 20. Krt20 deficiency compromised the mechanical load-bearing capacity of umbrella cells, triggering their adaptive expansion and activation of the mechanosensitive protein Yap. Furthermore, loss of Krt20 alters mechanical homeostasis in bladder tissue, stimulating TGF-β/Tgfbr1 signaling activation and subsequently resulting bladder fibrosis. Utilizing this model, we demonstrated a promising therapeutic potential by targeting Tgfbr1-dependent signaling pathway. FFPE snRNA sequencing identified that Alk4/5/7 inhibitor SB-431542 treatment partially rescued the fibrotic bladder microenvironment by attenuating TGF-β signaling derived. Collectively, we present a genetically defined LUTS-fibrosis model and elucidate a mechanotransduction-dependent pathogenic axis, offering innovative avenues for mechanism and therapy exploration.

## Highlights

• Keratin 20 forms a reticular cytoskeletal network in umbrella cells of mouse bladder
• Krt20 deficiency induces UAB-like LUTS and bladder fibrosis in mice
• TGF-β activation and mechanotransduction alteration associate with Krt20 deficiency
• TGF-β receptor inhibition alleviates bladder fibrosis in Krt20 deficient mice

## Graphical abstract

**Figure:**
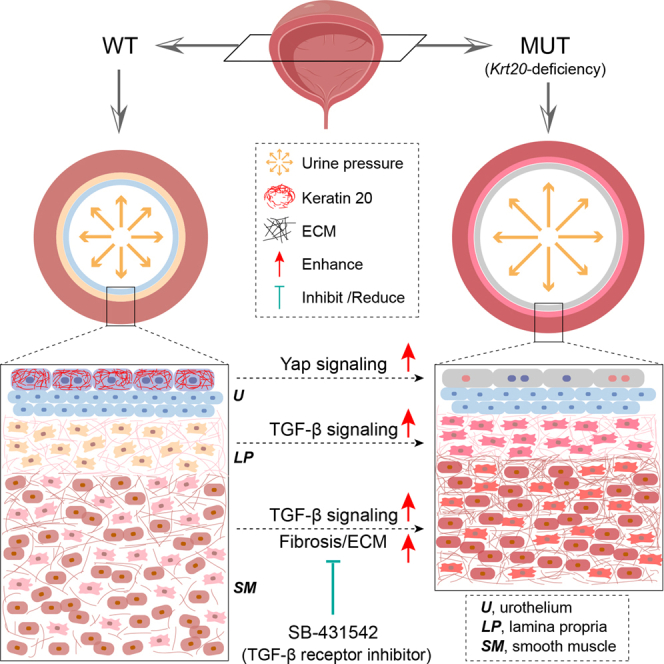

## Introduction

Lower urinary tract symptoms (LUTS) signify dysfunction in lower urinary tract organs representing a progressive disorder affecting approximately 2.3 billion people in worldwide.^1^^,^^2^^,^^3^ The etiology of LUTS is complex and multifactorial, including age, neurological degradation, inflammatory response and sex such as benign prostatic hyperplasia in men.^2^^,^^3^^,^^4^^,^^5^^,^^6^^,^^7^ Furthermore, low public awareness and undertreatment often allow LUTS to progress to severe lower urinary tract dysfunction (LUTD), potentially leading to urinary organ failure.^3^^,^^5^^,^^8^ Current clinical management primarily relies on neurotransmitter drugs and surgery,^2^^,^^9^^,^^10^ which often fail to halt disease progression and can be significant side effects.^2^^,^^9^ Therefore, there is an urgent need to explore the pathogenic mechanisms of LUTS and evaluate emerging precise therapies.

Bladder undergoes fibrotic remodeling were widely reported in many clinic LUTS patients those were diagnosed as neurogenic overactive bladder (OAB), underactive bladder (UAB), bladder outlet obstruction (BOO), and acute urinary retention (AUR).^11^^,^^12^^,^^13^^,^^14^^,^^15^^,^^16^ In addition, preclinical animal models also exhibited this pathological feature such as partial bladder outlet obstruction (pBOO),^17^^,^^18^^,^^19^^,^^20^ neurogenic UAB,^21^^,^^22^ radiation cystitis (RC),^23^^,^^24^ and ketamine-induced cystitis (KC).^25^^,^^26^ Bladder fibrosis usually contained the features of extracellular matrix (ECM) remodeling, elevated TGF-β1, and upregulated HIF-1α.^17^^,^^19^^,^^22^^,^^27^^,^^28^^,^^29^^,^^30^ Thus, targeting the fibrotic process may offer an innovative strategy for intervention of LUTS.

The urothelium acts as the first barrier of bladder protecting the underlying tissues from urine.^31^^,^^32^^,^^33^^,^^34^ To accommodate the bladder’s high compliance during urination cycle, the superficial umbrella cells are highly evolved against mechanical force and harmful urine penetration. The apical junctional complex (AJC), plays pivotal role in both mechanical balance and penetration barrier.^35^^,^^36^^,^^37^ AJC is elegantly assembled by tight junctions, adherens junctions, AJC-associated actin, desmosomes and associated keratin cytoskeleton to form a unique mesh-like network in umbrella cells,^35^^,^^36^^,^^37^ that harbors fusiform vesicles (DFVs) and responds to surface area dynamic adaptation.^38^^,^^39^^,^^40^ Although a few studies found AJC components E-cadherin and ZO-1 were reduced in pBOO animal models^17^^,^^20^ and BOO patients,^41^^,^^42^ however, the *in vivo* function evaluation of AJC components such as umbrella cell specifically expressed Krt20 are largely unexplored.

As an intermediate filament protein and AJC component, Keratin 20 is a hallmark of umbrella cells.^31^^,^^43^^,^^44^ In rats, KRT20 forms a luminal mesh and a deeper bundle layer visible only during bladder filling,^37^ hinting at a role in mechanoadaptation. Here, we generated *Krt20*-deficient mice and found *Krt20*-deficient leads to urinary retention, a UAB-like phenotype, and spontaneous bladder fibrosis. We further demonstrate that *Krt20*-deletion impairs mechanical integrity, activating Yap in umbrella cells and initiating a TGF-β1/Tgfbr1-dependent fibrotic cascade.

## Results

### Keratin 20-dependent cytoskeleton impairment induces adaptive expansion of umbrella cells

**Figure 1:**
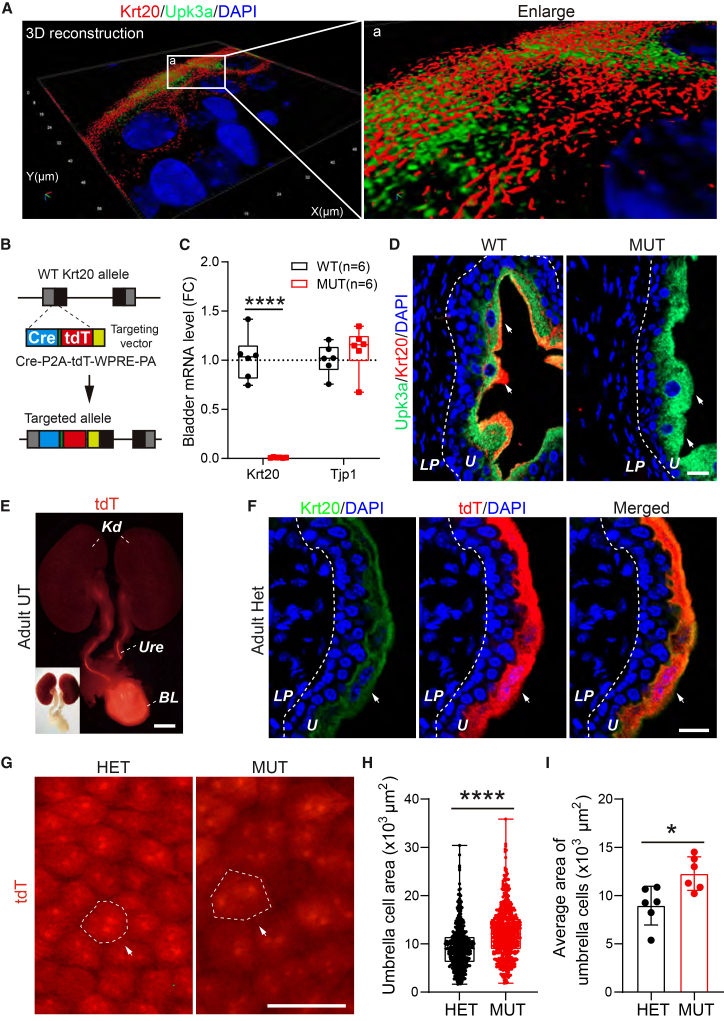
Krt20 deficient cytokeratin network leads to larger umbrella cell size (A) SMI imaged the mesh-like 3D-structure of Krt20-dependent cytokeratin network in umbrella cell apical surface. a. zoom-in region. (B) Schematic for generating Krt20-Cre-tdTomato mouse line. (C) RT-qPCR analysis showed the mRNA levels of *Krt20* and *Tjp1* in the bladders of *Krt20* mutant mice. (*n* = 6 mice for each group). Data are represented as mean ± SEM. Unpaired Student’s *t* test, ∗∗∗∗, *p* < 0.0001. (D) *Krt20* mutant mice lost KRT20 expression. Scale bar, 20 μm. U, Urothelium; LP, lamina propria. (E and F) Krt20-tdTomato *in situ* tracing shown that KRT20 protein restricted in urinary tract (E) and enriched in umbrella cell (F). Scale bar in (E), 2 mm. Scale bar in (F), 20 μm. BL, bladder; Ure, ureter; Kd, kidney, U, urothelium; LP, lamina propria. (G–I) Micro-dissected urothelium image (G) shown larger umbrella cell size (H and I) in *Krt20* mutant mice. (*n* = 6 mice for each group). Scale bars, 200 μm. For (H), each dot represents an umbrella cell. Data are represented as mean ± SEM. Unpaired Student’s *t* test, ∗, *p* < 0.05, ∗∗∗∗, *p* < 0.0001.

To characterize the organization pattern of Krt20 in mouse umbrella cells, we performed super-resolution imaging and 3D reconstruction, revealing that Krt20 assembles into a mesh-like cytoskeletal network in umbrella cells (Figure 1A). To investigate the function of Krt20, we generated a *Krt20*-Cre-tdTomato knock-in mouse line by inserting Cre-2A-tdTomato-Wpre-pA into the upstream of translation start site of exon 1 (Figure 1B). This model allows for *Krt20* knockout, Krt20 lineage tracing, and Krt20-driven Cre recombination. RT-qPCR and immunofluorescence confirmed mutant mice completely ablated *Krt20* in both mRNA and protein levels (Figures 1C and 1D). *Krt20* deficiency did not significantly affect Mendelian distribution, body weight, and lifespan (Figures S1A–S1C). As expected, tdTomato tracing *in situ* was restricted to the urinary system (Figure 1E) and highly co-localized with Krt20 antibody staining in umbrella cells (Figure 1F).

As keratin 20 dominantly expressed in umbrella cells, we firstly assessed the impact of Krt20 loss in umbrella cells. Using tdTomato as a tracer, we found that *Krt20* deficiency led to significantly increase the size of umbrella cell (Figures 1G–1I). This enlargement was independent of aberrant cell proliferation or division, as indicated by the lack of significant differences in Ki67 and pHH3 positive cells within both the urothelium and smooth muscle (SM) (Figures S1D–S1F). These findings indicate that the Krt20-dependent cytoskeleton network contributes to mechanical support. Immunofluorescence for urothelial markers (Krt20, Upk3A, Krt5, Krt8, and Krt15) and RT-qPCR analysis of differentiation markers (*Krt5*, *Krt7*, *Krt8*, *Krt18*, *Krt19*, *Tjp1*, *Upk1a*, *Upk1b*, *Upk2*, and *Upk3b*) revealed no significant alterations in cell layer distribution, proportions, or differentiation status in *Krt20*-deficient mice (Figures S2A–S2D). Umbrella cells in mutants retained their characteristic large, sometimes binucleated, morphology and expression of Upk3A and Krt8 (Figures 1D, S2A, and S2B). Thus, Krt20 loss specifically alters the mechanical properties of umbrella cells without affecting urothelial differentiation.

### Krt20-deficient mice phenocopied clinical urinary retention and UAB

**Figure 2:**
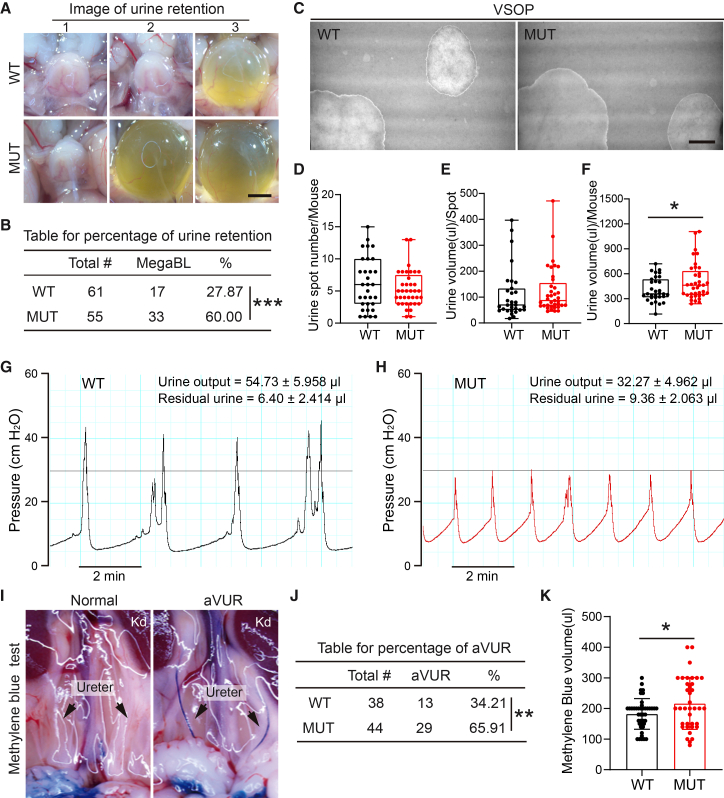
Krt20 deficient mice display urinary retention and underactive bladder phenotypes (A) Representative images of urinary retention bladder in *Krt20* mutant mice. Scale bars, 2 mm. (B) The frequency table for the urinary retention. Chi-square test, ∗∗∗, *p* < 0.001. (C) Representative images of VSOP detection within 2h of *Krt20* mutant mice. Scale bars, 2 cm. (D–F) Urine spot number (D), urine volume (E) and total urine volume (F) analysis for the mouse VSOP assay. (WT, *n* = 31, MUT, *n* = 37). Data are represented as mean ± SEM. Unpaired Student’s *t* test, ∗, *p* < 0.05. (G and H) Urodynamic analysis of wild-type (G) and *Krt20* mutant mice(H). (I) Representative images of artificial vesicoureteral reflux (aVUR). (J) The frequency table for the artificial vesicoureteral reflux (aVUR). Chi-square test, ∗∗, *p* < 0.01. (K) Maximal volume was measured by filling of methylene blue solution into bladder under anesthesia. (WT, *n* = 42, MUT, *n* = 37). Data are represented as mean ± SEM. Unpaired Student’s *t* test, ∗, *p* < 0.05.

Unexpectedly, we observed a significantly higher incidence of urinary retention in adult *Krt20*-deficient mice during euthanasia (Figures 2A and 2B), with a trend toward larger retained volume of bladder (Figure 2A). Clinical urinary retention can lead to compensatory bladder hyperplasia, vesicoureteral reflux (VUR), and incontinence.^29^^,^^30^^,^^45^ However, *Krt20*-deficient mice showed no significant changes in bladder or kidney weight (Figures S3A and S3B), or pathological signs of hydronephrosis (Figure S3C), suggesting the absence of severe compensatory bladder hyperplasia or renal damage.

Voiding spot of paper (VSOP) assay on filter papers revealed no significant difference in the number or average volume of urine spots over 2 h (Figures 2C–2E), but the total voided volume was significantly increased in *Krt20*-deficient mice (Figure 2F). Cystometry unveiled a UAB-like phenotype: voiding pressure dropped from ∼40 cm H_2_O to ∼30 cm H_2_O, voiding frequency increased approximately 2-fold (Figures 2G and 2H), voided volume significantly decreased (54.73 ± 5.958 μL vs. 32.27 ± 4.962 μL), and post-void residual urine significantly increased (6.40 ± 2.414 μL vs. 9.36 ± 2.063 μL) (Figures 2G and 2H). Furthermore, cystometry demonstrated that the micturition reflex initiation pressure in *Krt20* knockout mice (15–20 cm H_2_O) was higher than that in wild-type mice (10–15 cm H_2_O) (Figures 2G and 2H). This elevated pressure threshold for the micturition reflex indicates that *Krt20* deletion leads to impaired bladder sensation. Upon physically obstructing the urethra and infusing methylene blue, *Krt20*-deficient mice exhibited a significantly higher incidence of VUR (Figures 2I and 2J) and a trend toward significantly increased maximum bladder capacity (Figure 2K). These features align closely with clinical UAB,^13^^,^^14^ indicating the *Krt20*-deficient mouse as a preclinical UAB model.

### Keratin 20 deficiency activates TGF-β signaling and Yap in the urothelium

**Figure 3:**
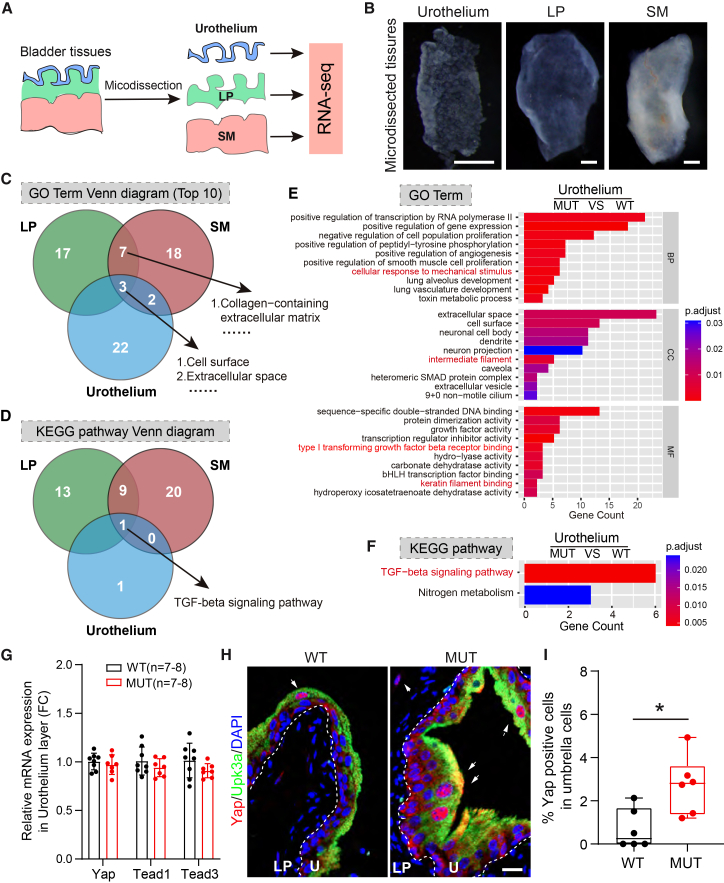
Krt20 deficiency activates TGF-β signaling and Yap in the urothelium (A) Schematic of bladder micro-dissection and experimental strategies. Group *N* = 2, three mice used grouped in each. (B) Representative images of urothelium, lamina propria, and smooth muscle layer tissue obtained from bladder microdissection. Scale bars, 0.5 mm. (C and D) Venn diagram analysis revealed common enriched GO terms (C) and KEGG signaling pathways (D) in urothelium, lamina propria, and smooth muscle layer. (E and F) Enriched GO terms (E) and KEGG signaling pathways (F) in urothelium. (G) RT-qPCR analysis was used to detect the expression of YAP signaling pathway-related genes in urothelium. Data are represented as mean ± SEM. (H and I) Representative images (H) and statistical analysis of YAP nuclear translocation (I) in urothelium. Scale bars, 20 μm. U, urothelium; LP, lamina propria. Data are represented as mean ± SEM. Unpaired Student’s *t* test, ∗, *p* < 0.05.

To characterize fibrotic molecular patterns in *Krt20*-deficient mice, we performed bulk RNA-seq on micro-dissected bladder layers^46^: urothelium (Ur), lamina propria (LP), and SM (Figures 3A and 3B). Analysis of differentially expressed genes (DEGs) and layer-specific markers^47^ (*Krt20*, *Krt8*, and *Upk3a* for Ur; *Col1a1*, *Col3a1*, and *Col7a1* for LP; *Acta2*, *Des*, and *Cnn1* for SM) confirmed successful dissection (Figures S4A–S4D). GO term analysis showed enrichment for “cell surface” and “extracellular space” were shared across three layers (Figure 3C). The Ur layer was uniquely enriched for “cellular response to mechanical stimulus”, “intermediate filament”, “keratin filament binding”, and “type I transforming growth factor β receptor binding” (Figure 3E), suggesting abnormal mechanical loading capacity of urothelial cells in the *Krt20*-deficient mice. Both LP and SM layers were enriched for “extracellular matrix” and “collagen-containing extracellular matrix” terms (Figures 3C, S4E, and S4F), suggesting collagen-dependent ECM remodeling occurred in the *Krt20*-deficient mice. KEGG pathway analysis identified the “TGF-β signaling pathway” as the only shared pathway across all three layers (Figures 3D, 3F, S5A, and S5B). Furthermore, KEGG pathway enrichment analysis identified significant enrichment of the “neuroactive ligand-receptor interaction” pathway in the bladder LP and SM layer (Figures S5A and S5B), indicating that Krt20 deletion results in aberrant neuronal signaling transmission.

Given the mechanosensitive terms was enriched in the Ur layer GO analysis and Yap usually used as a mechanosensory,^48^^,^^49^ we further investigated Yap nuclear translocation level. While *Yap*, *Tead1*, and *Tead3* mRNA levels were unchanged across layers (Figures 3G, S5C, and S5D), immunofluorescence revealed a significant increase in the proportion of umbrella cells with nuclear Yap localization in *Krt20*-deficient mice (Figures 3H and 3I), but not in muscle cells (Figures S5E and S5F). This indicates that Krt20 loss impaired mechanical load-bearing, leading to Yap activation in umbrella cells.

### Krt20 deficiency-induced LUTS is associated with TGF-β1 signaling and fibrosis

**Figure 4:**
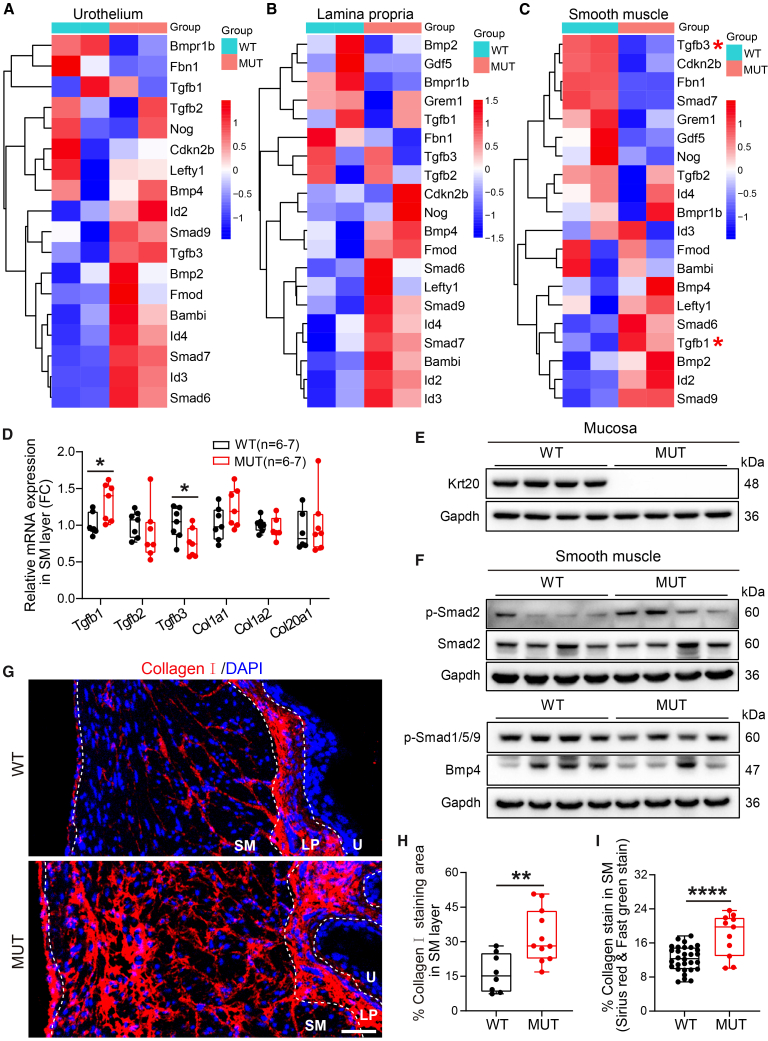
TGF-β1 signaling pathway was activated and bladder fibrosis occurred in Krt20 deficient mice (A–C) The cluster heatmap showed the DEGs enriched in TGF-β signaling pathway in bladder urothelium (A), lamina propria (B) and smooth muscle layers(C). (D) RT-qPCR analysis was used to further verify the TGF-β signaling pathway and downstream key genes in smooth muscle. Data are represented as mean ± SEM. Unpaired Student’s *t* test, ∗, *p* < 0.05. (E) Keratin Krt20 was completely absent in the mucosa of mutant mice. (F) Protein expression and activation of TGF-β signal transducer in smooth muscle layer of mutant mice. (G and H) Representative images of type I collagen immunofluorescence staining (E) and the proportion of stained area in the smooth muscle layer (F). (WT, *n* = 8, MUT, *n* = 11). Scale bars, 50 μm. Data are represented as mean ± SEM. Unpaired Student’s *t* test, ∗∗, *p* < 0.01. U, Urothelium; LP, lamina propria; SM, smooth muscle. (I) The proportion of total collagen analysis in smooth muscle layer. (WT, *n* = 30, MUT, *n* = 11). Data are represented as mean ± SEM. Unpaired Student’s *t* test, ∗∗∗∗, *p* < 0.0001.

To examine TGF-β signaling and ECM remodeling processes in mechanical stretching bladder, we visualized TGF-β pathway gene expression in three layers. In Ur and LP layers, TGF-β signaling associated transcripts *(Bmp4*, *Smad6*, *Smad7*, *Smad9*, *Id2*, *Id3*, and *Id4*) were generally upregulated (Figures 4A and 4B). However, SM layer exhibited a distinct pattern: When *Tgfb1*, *Bmp4*, *Smad6*, *Smad9*, and *Id2* were upregulated, while *Tgfb3*, *Smad7*, *Fbn1*, and *Gdf5* were downregulated (Figure 4C). This opposing pattern between *Tgfb1* (up) and *Tgfb3* (down) in the SM layer was confirmed by RT-qPCR (Figure 4D). No significant transcriptional changes were found in the LP (Figure S6A). To examine the activation of key transduction factors in the TGF-β signaling pathway, we performed microdissection to isolate the bladder mucosa and SM. We found that in the bladder SM of *Krt20* knockout mice, the TGF-β/Smad2 axis exhibited features of activation, whereas the Bmp/Smad1/5/9 axis showed characteristics of suppression (Figures 4E, 4F, and S6F). While confirming the complete loss of Krt20 in the mucosal layer, we found no evidence of associated changes in those TGF-β pathway indicators (Figures 4E, S6B, and S6E).

In general, TGF-β signaling activation is highly correlated with tissue fibrosis.^50^^,^^51^^,^^52^ We further assessed collagen deposition in the bladder of *Krt20*-deficient mice. Immunofluorescence and quantification showed a significant increase of type I collagen coverage in the SM layer of *Krt20*-deficient mice (Figures 4G and 4H). Sirius red/fast green staining confirmed increased total collagen content (Figures 4I and S6C). RNA-seq data indicated that collagen genes were expressed lowly in the urothelium but abundantly and dysregulated in the LP and SM layers of mutants (Figure S6D). Thus, *Krt20* deficiency induces Tgfb1 signaling activation and fibrotic remodeling.

### Targeting Alk4/5/7 ameliorates bladder fibrosis

**Figure 5:**
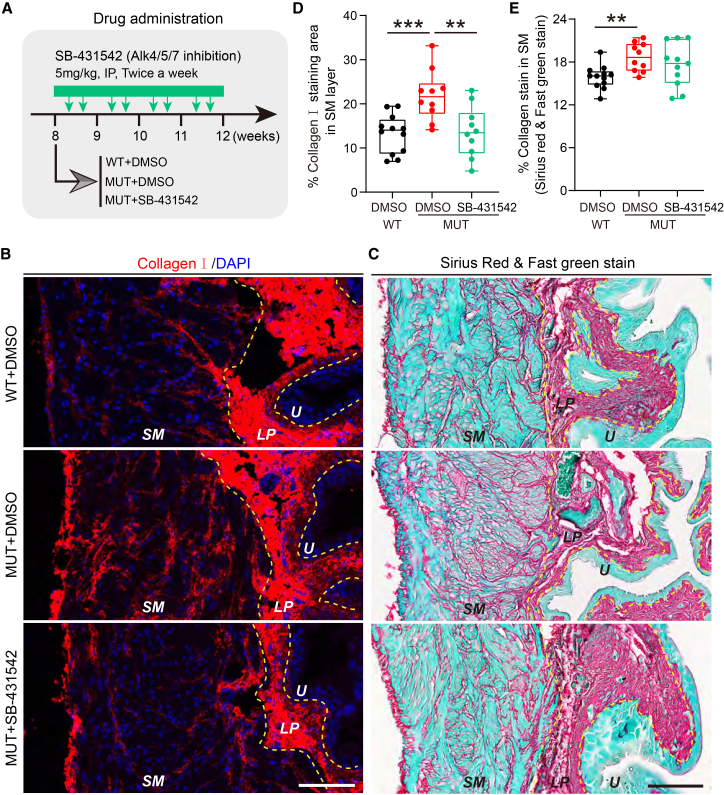
Alk4/5/7 inhibition partially alleviated mutant bladder fibrosis (A) Schematic of drug treatment strategy in Krt20 mutant mice. (B and C) Representative images of type I collagen (B) and total collagen staining (C) in Alk4/5/7 inhibitor SB-431542- or DMSO-treated mice. Scale bars, 100 μm. U, urothelium; LP, lamina propria; SM, smooth muscle. (D and E) Statistical results of the proportion of type I collagen (D) and total collagen (E) staining area in the smooth muscle layer. Each dot represents one mouse. Data are represented as mean ± SEM. Unpaired Student’s *t* test, ∗∗, *p* < 0.01, ∗∗∗, *p* < 0.001.

To test if fibrosis in this model is TGF-β signaling pathway-dependent, we treated *Krt20*-deficient mice with the Alk4/5/7 inhibitor SB-431542^53^ for 4 weeks (Figure 5A), as Tgfbr1 is designated as Alk5. Treatment showed no significant toxicity, as body, bladder, and kidney weights were unaffected (Figures S7A–S7C). As before, mutants showed increased type I collagen and total collagen, and SB-431542 treatment significantly reduced type I collagen levels to near-normal and showed a trend toward reducing total collagen (Figures 5B–5E).

The antifibrotic effect of SB-431542 exhibited sexual dimorphism pattern. In females, the inhibitor significantly reduced both type I collagen and total collagen (Figures S7D and S7E). In males, it significantly reduced type I collagen but not total collagen (Figures S7F and S7G). However, baseline *Tgfb1* expression in the bladder LP was lower in wild-type females than males (Figures S7H and S7I), this trend maintained in mutants (Figures S7J and S7K). This differential baseline may contribute to the dimorphic drug response.

### FFPE snRNA-seq reveals Alk4/5/7 inhibition partially rescues the fibrotic microenvironment by alleviating TGF-β signaling

**Figure 6:**
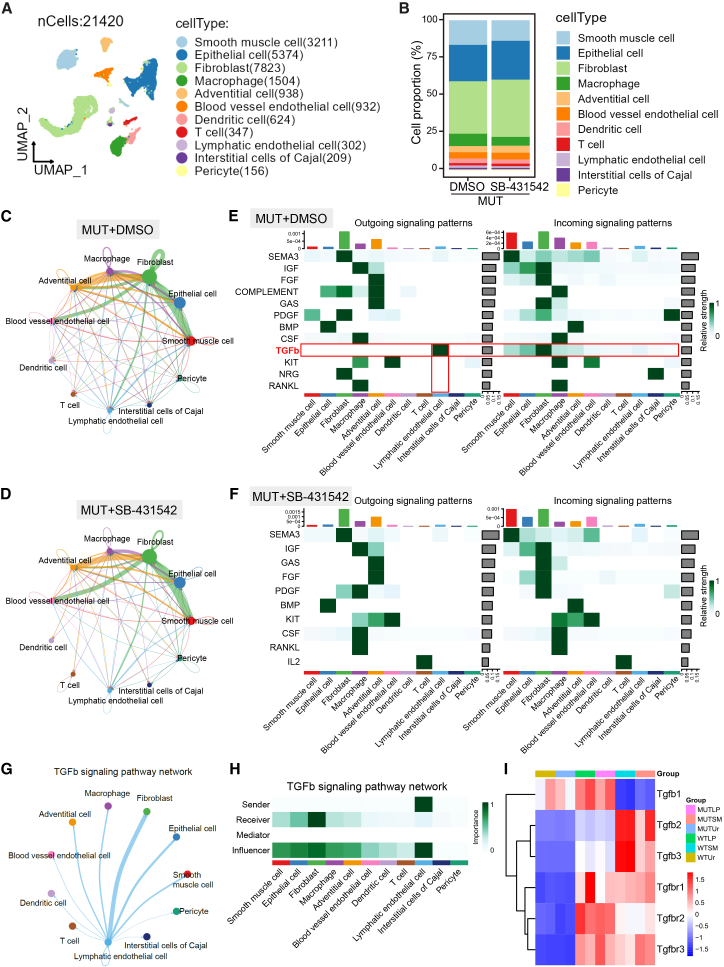
Alk4/5/7 inhibition affected cell-cell interaction among smooth muscle cells, fibroblasts, macrophages, and urothelial cells (A) UMAP analysis for cell clusters from paraffin single cell sequencing. (B) Cell fraction analysis for MUT+DMSO group and MUT+ SB-431542. (C and D) Cell-cell interaction/Cell chat analysis for MUT+DMSO group (C) and MUT+ SB-431542 group (D). (E and F) Incoming and outgoing signaling patterns analysis for MUT+DMSO group (E) and MUT+ SB-431542 group (F). (G and H) Analysis of TGF-β-mediated intercellular crosstalk (CellChat). (I) Heatmap shows transcriptional expression levels of TGF-β signaling ligands and receptors in bladder tissue layers.

Fibrosis usually remodeled local tissue microenvironment.^50^^,^^51^^,^^52^^,^^54^ To explore the pharmacological mechanism in females, we performed formalin-fixed paraffin-embedded (FFPE) single-nuclear RNA sequencing on bladders from *Krt20* mutant mice with or without SB-431542. We analyzed 21,420 nuclei from 11 specimens, identifying 11 cell types (Figure 6A). Cell proportion analysis revealed significant alterations in the proportions of SM cells, epithelial cells, fibroblasts, and macrophages following SB-431542 intervention, characterized by a decrease in SM cells and macrophages and a marked increase in fibroblasts and epithelial cells (Figure 6B). Cell-cell interaction analysis (CellChat) indicated that the predominant interactions occurred among these four cell types (Figures 6C and 6D). Furthermore, Alk4/5/7 inhibition reduced the overall strength and complexity of intercellular communication and significantly attenuated/blocked the enhanced TGF-β signaling in the bladders of *Krt20*-deficient mice (Figures 6E, 6F, S8A, and S8B).

To investigate the cellular origin and precise mechanism of action of TGF-β signaling, we performed an in-depth analysis of the FFPE snRNA-seq data and found that detectable TGF-β signaling originates predominantly from lymphatic endothelial cells (LECs) (*Lyve1*^+^*Prox1*^+^*Pecam1*^+^*Flt4*^+^) (Figures 6G and 6H). While this signaling influences nearly all cell types within the tissue, its primary functional target appears to be fibroblasts (Figures 6G and 6H). Furthermore, re-analysis of bulk RNA-seq data indicated that *Tgfb1* transcript levels are highest in the urothelium and LP, whereas its receptors (*Tgfbr1*, *Tgfbr2*, and *Tgfbr3*) are mainly expressed in the LP and SM layer (Figure 6I). Together, these findings demonstrate that Tgfb1 is produced by the urothelium and LP and acts primarily on the LP and SM.

To evaluate the intervention effects of SB-431542 on the tissue microenvironment, we performed a more in-depth analysis of the key cell populations following treatment. KEGG pathway enrichment analysis revealed that the “ECM-receptor interaction” signaling was attenuated in SM cells and macrophages following SB-431542 intervention (Figures S8C and S8F). In SM cells, SB-431542 intervention also attenuated “focal adhesion” and “regulation of actin cytoskeleton” signaling while concurrently restoring “circadian rhythm” and “vascular SM contraction” signaling (Figure S8C). In epithelial cells, SB-431542 intervention reduced “focal adhesion” signaling while concurrently restoring “retinol metabolism” and “Notch/Wnt signaling pathway” signaling (Figure S8D). In fibroblasts, SB-431542 intervention attenuated “regulation of actin cytoskeleton” signaling while concurrently restoring “ECM-receptor interaction”, “focal adhesion”, and “circadian rhythm” signaling (Figure S8E). In macrophages, SB-431542 intervention reduced “focal adhesion” and “vascular SM contraction” signaling while concurrently restoring “phagosome” and “ErbB signaling pathway” signaling (Figure S8F). In summary, the Alk4/5/7 inhibitor SB-431542 alleviates fibrosis primarily by remodeling the intercellular signaling network within major cell types in bladder tissue.

## Discussion

As an intermediate filament protein, Keratin 20 is a unique molecular marker for urothelial umbrella cells^31^^,^^43^^,^^44^ and it also serves as an indicator for molecular subtyping and prognostic assessment in bladder cancer.^55^^,^^56^^,^^57^ To date, only one study has described the assembly of Krt20 into a luminal mesh and a deeper bundle layer in rat umbrella cells, with the bundle layer being unique to the bladder filling phase.^37^ However, the functional role of Krt20 in the bladder remained unexplored. Our study confirms the mesh-like cytoskeletal structure of Krt20 in mouse umbrella cells and, through the *Krt20*-deficient mouse model, provides functional evidence that Krt20 is required for mechanical load-bearing capacity of umbrella cells and it protects the bladder from mechano-induced fibrosis. Loss of *Krt20* impaired this capacity and increased mechanical stretch in umbrella cells and entire bladder, ultimately initiating a Tgfb1/Tgfbr1 signaling-driven fibrotic program. This positions the urothelium as an active mechanosensory layer whose structural integrity is crucial for maintaining bladder wall homeostasis.

Here, we discovered an innovative pathological mechanism of *Krt20*-deficient bladder fibrosis: Krt20 dependent-mechanotransduction-TGF-β axis. Our hypothesis based on following evidences: (1) *Krt20*-deficient bladder elevated *p*-Smad2, while TGF-β receptor inhibitor SB-431542 significantly ameliorated bladder fibrosis; (2) *Krt20*-deficient bladder elevated yap nuclear translocation, and Yap inhibitor Verteporfin treatment has a decrease tread of bladder fibrosis as well (data not shown). (3) Fibrosis driven factor- TGF-β likely originates from bladder lymphatic endothelial cells (LECs), which was still debated in other fibrotic tissues.^58^^,^^59^

Clinically, UAB can be further categorized into three primary types: myogenic, neurogenic, and idiopathic.^14^^,^^60^ UAB often arises from multiple pathological processes and typically presents as a mixed entity. In our *Krt20*-deficient mouse model of UAB/LUTS, we observed that the threshold pressure for initiating the micturition reflex ranged between 15 and 20 cm H_2_O in *Krt20* knockout mice, whereas it was between 10 and 15 cm H_2_O in wild-type mice. This elevated pressure threshold indicates impaired bladder sensation in *Krt20*-deficient mice. Concurrently, KEGG pathway enrichment analysis of bulk RNA-seq data revealed significant enrichment of the “neuroactive ligand-receptor interaction” pathway within the bladder LP and SM layers, suggesting aberrant neuronal signaling. Therefore, we propose that the *Krt20* knockout mouse model likely recapitulates a neurogenic phenotype.

Our *Krt20*-deficient bladder fibrosis mouse model has certain advantages for drug development and evaluation. First, it offers a stable, non-surgical alternative to existing LUTS/fibrosis models that require complex interventions,^17^^,^^18^^,^^19^^,^^20^^,^^21^^,^^22^^,^^23^^,^^24^^,^^25^^,^^26^ making it particularly suitable for scalable drug screening. Indeed, we provide preclinical proof-of-concept that targeting the TGF-β signaling-dependent pathway can attenuate fibrosis, highlighting a promising therapeutic strategy. Furthermore, we also observed a sexual dimorphism in drug response in bladder fibrosis which underscores the importance of considering sex as a biological variable in designing anti-fibrotic therapies, a notion supported by studies in other organ systems.^61^^,^^62^^,^^63^^,^^64^ This systematic phenomenon is unlikely observed in cell level drug screen system.

Current LUTS treatments are often insufficient.^2^^,^^9^^,^^10^ The convergence of fibrosis across diverse LUTS types argues for targeting this common pathological process. Our work, along with a recent study showing that the anti-fibrotic drug pirfenidone improves voiding in a UAB model neurogenic UAB,^22^ strongly supports the feasibility and potential of repurposing anti-fibrotic agents for LUTS. Combining such agents with existing neuromodulatory approaches could represent a transformative multi-hit strategy, yet it requires further study.

In summary, we hypothesize Krt20 as a key guardian of urothelial mechanical function and demonstrate its critical role in preventing a mechano-pathological cascade leading to bladder fibrosis and certain UAB syndrome.

### Limitations of the study

Limitations include but not limit as following: first, we were unable to obtain clinical samples of UAB to establish the relevance between KRT20 and UAB. Second, we provided *Krt20* dependent-mechanotransduction-TGF-β axis for bladder fibrosis pathogenesis yet we did not exclude other possibilities. Third, although *Krt20* expression is restricted to umbrella cell in adult bladder, the urothelium-specific conditional knockout of *Krt20* would enhance the causable link. Lastly, although LECs likely triggers bladder fibrosis in our mutant yet further lineage deletion or critical gene conditional knockout would strengthen our hypothesis.

## Resource availability

### Lead contact

Inquiries for additional information or requests related to experimental details and reagents should communicate with the lead contact, Chunming Guo (chunmingguo@ynu.edu.cn).

### Materials availability

This study did not generate new unique reagents.

### Data and code availability

#### Data

• Micro-dissected bladder layer Bulk RNA-seq and FFPE snRNAseq raw data have been deposited at NGDC (NGDC: CRA025566 https://ngdc.cncb.ac.cn/gsa/search?searchTerm=CRA025566, and CRA029729 https://ngdc.cncb.ac.cn/gsa/search?searchTerm=CRA029729).

#### Code

• This paper does not report the original code.

#### Additional information

• Any additional information required to reanalyze the data reported in this paper is available from the lead contact upon request.

## Acknowledgments

This work was supported by the 10.13039/501100001809National Natural Science Foundation of China no. 82460142 to C.M.G., the Xingdian talent support program of Yunnan Province to C.M.G., Guangdong Hybribio Biotech Funding no. H20230314, H20230311, and H20230313 to C.M.G., and NHC Key Laboratory of Healthy Birth and Birth Defect Prevention in Western China, no. 2024XBYSKF010 to C.M.G. We thank Dong Li (Tsinghua University) and Lan Mei (ZEISS) for SIM image processing support. We thank the M20 Genomics team for their technical support with FFPE snRNA-seq and for assistance with bioinformatic analyses.

## Author contributions

C.G. conceived and supervised the project, also provided the funding support. J.J. conducted project and wrote the draft of manuscripts. Jiasheng Chen carried out living mice urodynamic test, Y.B., T.H., C.M., and X.D. participated mice experiments, R.G. carried out FFPE snRNA-seq experiment, H.Z., W.M., J.H., Jingjie Chen, Jiehui Chen, F.Y., X.L., Y.C.H., and F.C. participated in data analysis, image preparation and manuscript correction.

## Declaration of interests

The authors declare no competing interests.

## STAR★Methods

### Key resources table

**Table:** 

REAGENT or RESOURCE	SOURCE	IDENTIFIER
**Antibodies**		

Mouse monoclonal Anti-Krt20 [Clone Ks20.8]	Dako	Cat# M7019; RRID:AB_2133718
Rabbit monoclonal Anti-Krt20 [Clone D9Z1Z]	Cell Signaling Technology	Cat# 13063; RRID:AB_2798106
Rabbit polyclonal Anti-Krt5	Abcam	Cat# ab53121; RRID:AB_869889
Mouse monoclonal Anti-Upk3a [AU1]	provided by Dr. T.T. Sun	N/A
Rat monoclonal Anti-Krt8/TROMA-I [Clone SP2/0]	DSHB	Cat# AB_531826; N/A
Rabbit polyclonal Anti-Krt15	ABclonal	Cat# A2660; RRID:AB_2764526
Rabbit monoclonal Anti-Collagen I [Clone EPR24331-53]	Abcam	Cat# ab270993; RRID:AB_2927551
Rabbit monoclonal Anti-Yap [Clone D8H1X]	Cell Signaling Technology	Cat# 14074; RRID:AB_2650491
Rabbit polyclonal Anti-Ki67	Servicebio	Cat# GB111141; RRID:AB_3096315
Mouse monoclonal Anti-Phospho-Histone H3 (Ser10) (Clone 3H10)	Sigma-Aldrich	Cat# 05-806; RRID:AB_310016
Mouse monoclonal Anti-SMAD2 (Clone L16D3)	Cell Signaling Technology	Cat# 3103; RRID:AB_490816
Rabbit monoclonal Anti-Phospho-SMAD2 (Ser465/Ser467) (Clone E8F3R)	Cell Signaling Technology	Cat# 18338; RRID:AB_2798798
Rabbit Monoclonal Anti-Phospho-Smad1/5/9 (S463/S465/S467) (Clone JE59-46)	HUABIO	Cat# HA722566; N/A
Mouse monoclonal Anti-BMP4(Clone 6B7)	Cell Signaling Technology	Cat# 4680; RRID:AB_2274651
Mouse monoclonal Anti-GAPDH (Clone 1E6D9)	Proteintech	Cat# 60004-1-Ig; RRID:AB_2107436
Anti-mouse IgG, HRP-linked Antibody	Cell Signaling Technology	Cat# 7076; RRID:AB_330924
Anti-rabbit IgG, HRP-linked Antibody	Cell Signaling Technology	Cat# 7074; RRID:AB_2099233
Cy™2 AffiniPure™ Donkey Anti-Mouse IgG (H+L)	Jackson ImmunoResearch	Cat# 715-225-150; RRID:AB_2340826
Cy™3 AffiniPure™ Donkey Anti-Rabbit IgG (H+L)	Jackson ImmunoResearch	Cat# 711-165-152; RRID:AB_2307443
Cy™2 AffiniPure™ Donkey Anti-Rat IgG (H+L)	Jackson ImmunoResearch	Cat# 712-225-150; RRID:AB_2340673

**Chemicals, peptides, and recombinant proteins**		

Paraformaldehyde	Sigma-Aldrich	Cat# 441244
O.C.T. Compound	SAKURA	Cat# 4583
Paraffin wax	CITOTEST	Cat# 80200-0016
Sucrose	Sangon Biotech	Cat# A610498
Isoflurane	RWD	Cat# R510-22
Sodium citrate dihydrate	Sigma-Aldrich	Cat# W302600
Triton X-100	Sangon Biotech	Cat# A600198
Animal-Free Blocking Solution (5X)	Cell Signaling Technology	Cat# 15019
DAPI	Invitrogen	Cat# D3571
Fluoromount-G®	Southern Biotech	Cat# 0100-01
TRIzol™ Reagent	Invitrogen	Cat# 15596026
Trichloromethane (AR,99%)	KESHI	CAS# 67-66-3
Isopropanol	Sangon Biotech	Cat# A507048
UltraPure™ DNase/RNase-Free Distilled Water	Invitrogen	Cat# 10977023
DMSO	Sigma-Aldrich	Cat# D2650
SB-431542	MedChemExpress	Cat# HY-10431
Hematoxylin staining solution	Servicebio	Cat# G1005-1
Eosin staining solution (alcohol-soluble type)	Servicebio	Cat# G1005-2
Bouin’s solution	Sigma-Aldrich	Cat# HT10132
Fast green FCF	Sigma-Aldrich	Cat# F7252
Picrosirius Red Staining Solution	PHYGENE Scientifc	Cat# PH1098
Methylene Blue	Solarbio	Cat# M8030

**Critical commercial assays**		

HiScript III 1st Strand cDNA Synthesis Kit (+gDNA wiper)	Vazyme	Cat# R312-02
NovoStart® SYBR High-Sensitivity qPCR SuperMix	Novoprotein	Cat# E099
2X Rapid Taq Master Mix	Vazyme	Cat# P222-03
M&R HRP/DAB Detection IHC Kit	Vazyme	Cat# HC301-02
SuperSignal. West Pico PLUS Chemiluminescent Substrate	Thermo Scientific	Cat# 34580

**Deposited data**		

Bladder micro-dissected tissue RNA-seq GSA dataset	This paper	NGDC: CRA025566https://ngdc.cncb.ac.cn/gsa/search?searchTerm=CRA025566
FFPE snRNAseq raw data GSA dataset	This paper	NGDC: CRA029729https://ngdc.cncb.ac.cn/gsa/search?searchTerm=CRA029729

**Experimental models: Organisms/strains**		

Mouse: Krt20-Cre-tdTomato	This paper	Shanghai Model Organisms Center, Inc
Mouse: C57BL/6J	The Jackson Laboratory	JAX# 000664

**Oligonucleotides**		

See Table S1	N/A	N/A

**Software and algorithms**		

GraphPad Prism	GraphPad Software	Prism 9.0.0
Image J	National Institutes of Health	Image J 1.53a
ZEN	ZEISS	ZEN 2.3
Arivis Vision 4D software	ZEISS	Version 4.2.2
Adobe Illustrator	Adobe	Illustrator 2023
Adobe Photoshop	Adobe	Photoshop 2023
SRplot	N/A	https://www.bioinformatics.com.cn
ClusterProfiler software	Bioconductor	Version 4.18.4
LabChart 8 Reader	ADInstruments	Version 8.1.30

**Other**		

Filter paper	Solarbio	Cat# YA0166-1EA

### Experimental model and study participant details

#### Animals

All animal studies were performed according to protocols reviewed and approved by the Institutional Animal Care and Use Committee (IACUC) at Yunnan University: YNU20220176 and YNU20240858. All protocols for animal studies followed guidelines. The Krt20-Cre-tdTomato knock-in mouse line was generated by knocking Cre-2A-tdTomato-Wpre-pA before exon 1 of Krt20 gene (after 5′ UTR) by homologous recombination using CRISPR/Cas9. The Krt20-Cre-tdTomato lines were generated by Shanghai Model Organisms Center. C57BL/6J mice (JAX#000664) were obtained from the Jackson Laboratory. In all animal experiments, adult mice (8–9 weeks old) were used. Mice were grouped by genotype, and male and female mice were randomly unless necessary.

### Method details

#### Tissue preparation and histological analysis

For paraffin section sample preparation, bladder tissues were collected and fixed in 4% PFA(Sigma) overnight at 4°C and then dehydrated and embedded according to standard procedures. Paraffin sections were prepared to a thickness of 5 μm and collected on slides. For frozen section sample preparation, bladder tissues were collected and fixed in 4% PFA (Sigma) at 4°C for 2h. The bladder tissues were dehydrated in 30% sucrose at 4°C overnight and embedded in O.C.T. (SAKURA) at −80°C for 30 min. Frozen sections were prepared to a thickness of 10 μm and collected on slides. Standard hematoxylin and eosin staining (H&E) were used for histological analysis. Sirius Red & Fast green staining was performed as described previously.^65^ Briefly, after paraffin/O.C.T. (SAKURA) removal, tissue sections were incubated overnight at room temperature in Bouin’s solution (Sigma). After washing three times, the sections were incubated in 0.1% Fast Green FCF (Sigma) for 10min. Then, the slides were washed three times and incubated in Picrosirius Red Staining Solution (PHYGENE Scientifc) for 1min. Images were acquired with Slide Scan System (Teksqray SQS-1000) according to the instructions, and then Image J was used to calculate the Sirius Red staining area ratio for the analysis of total collagen content in bladder muscles.

#### Immunofluorescence staining

After removing paraffin/O.C.T. (SAKURA) from tissue sections, the paraffin sections need to be placed in 10 mM Sodium citrate dihydrate (Sigma) solution at pH=6 for hot antigen repair for 10min. Then, tissue sections were blocked in PBS containing 0.2%Triton X-100 and Animal-Free Blocking Solution (CST) for 1h, incubated with primary antibody overnight at 4 °C, and incubated with secondary antibody and DAPI(Invitrogen) for 1h at room temperature. Slides were observed with a ZEISS laser confocal microscope (LSM 800) according to the operating instructions. Antibodies used for immunofluorescent staining were listed in key resources table.

#### Immunohistochemistry

The protocol is essentially identical to that for immunofluorescence staining, with the addition of an endogenous peroxidase quenching step. Staining was performed using HRP-conjugated secondary antibodies (M&R HRP/DAB Detection IHC Kit), and images were acquired with a Slide Scan System (Teksqray SQS-1000) according to the instructions.

#### Western Blot

Proteins were extracted from microdissected bladder mucosa and smooth muscle using RIPA buffer with protease/phosphatase inhibitors. Protein concentration was measured by BCA assay (Enhanced BCA Protein Assay Kit). Lysates were denatured, separated on 4–20% Tris-Glycine gels, and transferred to PVDF membranes. After blocking with 5% Non-Fat Powdered Milk, membranes were incubated overnight with primary antibodies at 4°C, followed by HRP-conjugated secondary antibodies. Signals were detected by enhanced chemiluminescence (ECL) and imaged using a chemiluminescence system.

#### Multi-SIM imaging and 3D reconstruction

SIM images were acquired on a Multi-SIM (Multimodality Structured Illumination Microscopy) imaging system (NanoInsights-Tech Co., Ltd.) equipped with a 63 × 1.40NA oil objective (ZEISS Objective Plan-Apochromat 63x/1.4 Oil M27), and a Photometrics Kinetix camera. Zeiss arivis Vision 4D software (version 4.2.2) was employed for 3D visualization of images taken by Multi-SIM. Threshold-based segmentation was used to isolate Krt20 and Upk3a positive objects, which were enhanced by Surface rendering. Snapshots of 3D model with different orientation were generated. And the movies were recorded by rotating and moving 3D model in Arivis Vision 4D.

#### Imaging of organs, tissues and umbrella cells

Direct imaging of the urinary system, urinary retention bladder, and bladder tissue layers was performed using a stereo dissecting microscope (OLYMPUS SZX16). Here, we have introduced the imaging method of umbrella cells. Briefly, mouse bladders were obtained and quickly placed on slides, and 1-2 drops of cold autoclaves of PBS were added. Under stereo dissecting microscope, the bladder was cut into 2-3 pieces and dissected to obtain the mucosal layer. Finally, the umbrella cell layer of the mucosal layer was tiled upward, covered with a cover slip and gently pressed for fluorescence imaging.

#### VSOP analysis

VSOP was performed as described previously.^66^ Briefly, filter paper (Solarbio, Cat# YA0166-1EA) was placed at the bottom of boxes, Mice were placed individually in each box for 2h and the urine drops were collected onto the filter paper as voided stain. At the end the assay period, filter paper was retrieved and urine spots left behind were imaged under ultraviolet light. Image J was used to analyze the area of urine spots and count the number of urine spots. In addition, we drew a linear regression curve (y = 9.714x - 2.4111, R^2^ = 0.9964) based on the corresponding relationship between the standard amount of mouse urine and the area of urine spots, and calculated the urine volume of each urine spot.

#### Cystometry

Cystometry was performed as described previously.^66^^,^^67^^,^^68^ Briefly, under urethane (1.8 g/kg) anesthesia, the bladder and ureter were surgically exposed, and the ureter was ligated distally. Then, a polyethylene catheter (PE-50, Clay Adams) was inserted into the bladder from the bladder dome and connected to a pressure transducer and infusion pump (AD Instruments) via a three-way stopcock. Sterile PBS was instilled into the bladder through an infusion pump at a rate of 1.2ml/h, and pressure and voiding recordings were performed with a LabChart 8 system. At the end of the experiment, the mice were euthanized.

#### Bladder filling experiments

To examine the maximum bladder volume in Krt20-null mice, we performed bladder filling experiments using methylene blue solution. Briefly, mice were anesthetized with Isoflurane (RWD) and the surgical site was sterilized with 75% ethanol. Then, the abdominal cavity was opened to expose the bladder, and the outlet of the urethra was clamped with a hemostatic clamp. Methylene blue (Solarbio) solution (10mg/ml) was injected vertically (at a rate of ∼15s/200μl) into the bladder using a 26G-1mL syringe until solution could not expand bladder anymore. During this period, the volume of methylene blue injected was recorded. All mice were euthanized at endpoint.

#### Bladder micro-dissection

Bladder micro-dissection was performed as described previously.^46^ Based on this microdissection method, we successfully isolated the urothelium, lamina propria and smooth muscle layers of the mouse bladder. Briefly, mouse bladder was harvested and placed immediately in cold autoclaved PBS. Under stereo dissecting microscope (OLYMPUS SZX16), the bladder was trimmed to a long strip, and then the smooth muscle layer and mucosa were separated with finite forceps, and the lamina propria layer and urothelium layer of mucosa were separated with small-angle fine tweezers. Because mice have few bladder epithelial cells, we usually combined bladder epithelial cells from three mice as Mix samples for subsequent RT-qPCR and RNA-seq analyses.

#### RNA isolation, RNA-seq and RT-qPCR

TRIzol reagent (Invitrogen) was used to isolate total RNA from micro-dissected bladder urothelium, lamina propria and smooth muscle. RNA-seq (PE150) was performed by Annoroad Gene Technology using DNBSEQ-T7. Cutoff for differentially expressed genes (DEGs) was based on padj < 0.05 and more than 2-fold changes. Pathway analysis was done using the ClusterProfiler software package. Meanwhile, we also used the online tool SRplot to draw the cluster heatmap. For RT-qPCR experiments, mRNA was reverse-transcribed into cDNA using the HiScript III 1st Strand cDNA Synthesis Kit (+gDNA wiper) (Vazyme). RT-qPCR analyses were performed using NovoStart® SYBR High-Sensitivity qPCR SuperMix (Novoprotein) on a CFX96TM Real-Time System C1000 Touch detector (BioRad CFX Manager). Relative gene expression levels were normalized to the internal control Gapdh. Gene-specific primers were listed in Table S1.

#### Drug administration

To test pharmacological efficacy of Alk4/5/7 inhibitor SB-431542 (MCE) on bladder fibrosis, we injected SB-431542 intraperitoneally to intervene in the progression of bladder fibrosis. Briefly, Krt20 mutant mice were treated with SB-431542 at a dose of 5mg/kg twice a week. Control Krt20 mutant and WT mice were treated with the same dose of DMSO(Sigma). One month later, the mice bladder tissues were collected to evaluate the anti-fibrotic pharmacological activity of SB-431542 based on the degree of fibrosis.

#### FFPE snRandom-seq and data analysis

Formalin-fixed paraffin-embedded (FFPE) samples intervened with SB-431542 were cut from paraffin blocks with a size of 20 μm per section and 5 sections per sample. Five to six mouse bladder sections were mixed into groups. Then, the sequencing library was constructed according to the VITApilote high-throughput FFPE single-cell transcriptome kit (v1.7). Sequencing was performed using the Illumina novaseq Xplus sequencing platform with a sequencing length of PE150. We preprocessed the snRandom-seq data according to the procedures detailed in the snRandom-seq protocol previously published,^69^ and then performed Clustering, CellChat and KEGG Enrichment analysis.

### Quantification and statistical analysis

Statistical analysis was performed using GraphPad Prism 9, with statistical significance set as p < 0.05. Significance levels were defined as ∗, p < 0.05, ∗∗, p < 0.01, ∗∗∗, p < 0.001, ∗∗∗∗, p < 0.0001. Student’s t test was used to analyze datasets with normal distribution including RT-qPCR, proportion of cell type, cell size, urine spot volume, methylene blue volume and fibrosis area assessment. The Chi-square test was mainly used for urine retention frequency analysis. All experiments were repeated more than three times to ensure reproducibility. Data is reported in the form of box and whisker plots with individual data points.

## References

[1] Irwin D.E., Kopp Z.S., Agatep B., Milsom I., Abrams P. (2011). Worldwide prevalence estimates of lower urinary tract symptoms, overactive bladder, urinary incontinence and bladder outlet obstruction. BJU Int..

[2] Wei J.T., Dauw C.A., Brodsky C.N. (2025). Lower Urinary Tract Symptoms in Men. JAMA.

[3] Gibson W., Wagg A. (2017). Incontinence in the elderly, 'normal' ageing, or unaddressed pathology?. Nat. Rev. Urol..

[4] Vignozzi L., Gacci M., Maggi M. (2016). Lower urinary tract symptoms, benign prostatic hyperplasia and metabolic syndrome. Nat. Rev. Urol..

[5] Rodriguez-Nieves J.A., Macoska J.A. (2013). Prostatic fibrosis, lower urinary tract symptoms, and BPH. Nat. Rev. Urol..

[6] Zhang L., Zhu L., Xu T., Lang J., Li Z., Gong J., Liu Q., Liu X. (2015). A Population-based Survey of the Prevalence, Potential Risk Factors, and Symptom-specific Bother of Lower Urinary Tract Symptoms in Adult Chinese Women. Eur. Urol..

[7] Wang Y., Hu H., Xu K., Wang X., Na Y., Kang X. (2015). Prevalence, risk factors and the bother of lower urinary tract symptoms in China: a population-based survey. Int. Urogynecol. J..

[8] Chapple C., Castro-Diaz D., Chuang Y.-C., Lee K.-S., Liao L., Liu S.-P., Wang J., Yoo T.K., Chu R., Sumarsono B. (2017). Prevalence of Lower Urinary Tract Symptoms in China, Taiwan, and South Korea: Results from a Cross-Sectional, Population-Based Study. Adv. Ther..

[9] Michel M.C., Cardozo L., Chermansky C.J., Cruz F., Igawa Y., Lee K.-S., Sahai A., Wein A.J., Andersson K.-E., Daws L. (2023). Current and Emerging Pharmacological Targets and Treatments of Urinary Incontinence and Related Disorders. Pharmacol. Rev..

[10] de Groat W.C., Griffiths D., Yoshimura N. (2015). Neural Control of the Lower Urinary Tract. Compr. Physiol..

[11] Compérat E., Reitz A., Delcourt A., Capron F., Denys P., Chartier-Kastler E. (2006). Histologic Features in the Urinary Bladder Wall Affected from Neurogenic Overactivity—A Comparison of Inflammation, Oedema and Fibrosis With and Without Injection of Botulinum Toxin Type A. Eur. Urol..

[12] Yang X., Pu Q., Wen Y., Zhao Y., Wang J., Xu P., Ma Y., Liu E., Lv L., Wen J.G. (2022). Activation of the TGF-β1/Smads/α-SMA pathway is related to histological and functional changes in children with neurogenic bladder. Sci. Rep..

[13] Taylor J.A., Kuchel G.A. (2006). Detrusor Underactivity: Clinical Features and Pathogenesis of an Underdiagnosed Geriatric Condition. J. Am. Geriatr. Soc..

[14] Wang J., Ren L., Liu X., Liu J., Ling Q. (2023). Underactive Bladder and Detrusor Underactivity: New Advances and Prospectives. Int. J. Mol. Sci..

[15] Collado A., Batista E., Gelabert-Más A., Corominas J.M., Arañó P., Villavicencio H. (2006). Detrusor quantitative morphometry in obstructed males and controls. J. Urol..

[16] Deveaud C.M., Macarak E.J., Kucich U., Ewalt D.H., Abrams W.R., Howard P.S. (1998). Molecular analysis of collagens in bladder fibrosis. J. Urol..

[17] Iguchi N., Hou A., Koul H.K., Wilcox D.T. (2014). Partial Bladder Outlet Obstruction in Mice May Cause E-Cadherin Repression through Hypoxia Induced Pathway. J. Urol..

[18] Wang N., Lu L., Cao Q.f., qian S., Ding J., Wang C., Duan H., Shen H., Qi J. (2021). Partial inhibition of activin receptor-like kinase 4 alleviates bladder fibrosis caused by bladder outlet obstruction. Exp. Cell Res..

[19] Wiafe B., Kadam R., Metcalfe P.D. (2020). Intraperitoneal administration of mesenchymal stem cells is effective at mitigating detrusor deterioration after pBOO. Am. J. Physiol. Renal Physiol..

[20] Zhang Z., Zhanghuang C., Mi T., Jin L., Liu J., Li M., Wu X., Wang J., Li M., Wang Z. (2023). The PI3K-AKT-mTOR signaling pathway mediates the cytoskeletal remodeling and epithelial-mesenchymal transition in bladder outlet obstruction. Heliyon.

[21] Weyne E., Dewulf K., Deruyer Y., Rietjens R., Everaerts W., Bivalacqua T.J., De Ridder D., Van der Aa F., Albersen M. (2018). Characterization of voiding function and structural bladder changes in a rat model of neurogenic underactive bladder disease. Neurourol. Urodyn..

[22] Ko I.-G., Hwang L., Jin J.-J., Kim S.-H., Kim C.-J., Choi Y.H., Kim H.Y., Yoo J.M., Kim S.J. (2024). Pirfenidone improves voiding function by suppressing bladder fibrosis in underactive bladder rats. Eur. J. Pharmacol..

[23] Zwaans B.M.M., Wegner K.A., Bartolone S.N., Vezina C.M., Chancellor M.B., Lamb L.E. (2020). Radiation cystitis modeling: A comparative study of bladder fibrosis radio-sensitivity in C57BL/6, C3H, and BALB/c mice. Physiol. Rep..

[24] Zwaans B.M.M., Grobbel M., Carabulea A.L., Lamb L.E., Roccabianca S. (2022). Increased extracellular matrix stiffness accompanies compromised bladder function in a murine model of radiation cystitis. Acta Biomater..

[25] Juan Y.-S., Lee Y.-L., Long C.-Y., Wong J.-H., Jang M.-Y., Lu J.-H., Wu W.-J., Huang Y.-S., Chang W.-C., Chuang S.-M. (2015). Translocation of NF-κB and Expression of Cyclooxygenase-2 Are Enhanced by Ketamine-Induced Ulcerative Cystitis in Rat Bladder. Am. J. Pathol..

[26] Wang J., Chen Y., Gu D., Zhang G., Chen J., Zhao J., Wu P. (2017). Ketamine-induced bladder fibrosis involves epithelial-to-mesenchymal transition mediated by transforming growth factor-β1. Am. J. Physiol. Renal Physiol..

[27] Chen Y., Ma Y., He Y., Xing D., Liu E., Yang X., Zhu W., Wang Q., Wen J.G. (2021). The TGF-β1 pathway is early involved in neurogenic bladder fibrosis of juvenile rats. Pediatr. Res..

[28] Li Q., Hong Y., Chen J., Zhou X., Tian X., Yu Y., Shen L., Long C., Cai M., Wu S., Wei G. (2022). Hypoxia-Induced HIF-1α Expression Promotes Neurogenic Bladder Fibrosis via EMT and Pyroptosis. Cells.

[29] Kim S.J., Kim J., Na Y.G., Kim K.H. (2021). Irreversible Bladder Remodeling Induced by Fibrosis. Int. Neurourol. J..

[30] Fusco F., Creta M., De Nunzio C., Iacovelli V., Mangiapia F., Li Marzi V., Finazzi Agrò E. (2018). Progressive bladder remodeling due to bladder outlet obstruction: a systematic review of morphological and molecular evidences in humans. BMC Urol..

[31] Wiessner G.B., Plumber S.A., Xiang T., Mendelsohn C.L. (2022). Development, regeneration and tumorigenesis of the urothelium. Development.

[32] Balsara Z.R., Li X. (2017). Sleeping beauty: awakening urothelium from its slumber. Am. J. Physiol. Renal Physiol..

[33] Dalghi M.G., Montalbetti N., Carattino M.D., Apodaca G. (2020). The Urothelium: Life in a Liquid Environment. Physiol. Rev..

[34] Andersson K.E., Arner A. (2004). Urinary bladder contraction and relaxation: Physiology and pathophysiology. Physiol. Rev..

[35] Carattino M.D., Prakasam H.S., Ruiz W.G., Clayton D.R., McGuire M., Gallo L.I., Apodaca G. (2013). Bladder filling and voiding affect umbrella cell tight junction organization and function. Am. J. Physiol. Renal Physiol..

[36] Eaton A.F., Clayton D.R., Ruiz W.G., Griffiths S.E., Rubio M.E., Apodaca G. (2019). Expansion and contraction of the umbrella cell apical junctional ring in response to bladder filling and voiding. Mol. Biol. Cell.

[37] Ruiz W.G., Clayton D.R., Parakala-Jain T., Dalghi M.G., Franks J., Apodaca G. (2024). The rat bladder umbrella cell keratin network: Organization, dependence on the plectin cytolinker, and responses to bladder filling. Mol. Biol. Cell.

[38] Truschel S.T., Wang E., Ruiz W.G., Leung S.M., Rojas R., Lavelle J., Zeidel M., Stoffer D., Apodaca G. (2002). Stretch-regulated exocytosis/endocytosis in bladder umbrella cells. Mol. Biol. Cell.

[39] Khandelwal P., Ruiz W.G., Apodaca G. (2010). Compensatory endocytosis in bladder umbrella cells occurs through an integrin-regulated and RhoA- and dynamin-dependent pathway. Embo J..

[40] Wu X.-R., Kong X.-P., Pellicer A., Kreibich G., Sun T.-T. (2009). Uroplakins in urothelial biology, function, and disease. Kidney Int..

[41] Jiang Y.-H., Lee C.-L., Kuo H.-C. (2016). Urothelial Dysfunction, Suburothelial Inflammation and Altered Sensory Protein Expression in Men with Bladder Outlet Obstruction and Various Bladder Dysfunctions: Correlation with Urodynamics. J. Urol..

[42] Jiang Y.-H., Kuo H.-C. (2017). Urothelial Barrier Deficits, Suburothelial Inflammation and Altered Sensory Protein Expression in Detrusor Underactivity. J. Urol..

[43] Coulombe P.A., Wong P. (2004). Cytoplasmic intermediate filaments revealed as dynamic and multipurpose scaffolds. Nat. Cell Biol..

[44] Snider N.T., Omary M.B. (2014). Post-translational modifications of intermediate filament proteins: mechanisms and functions. Nat. Rev. Mol. Cell Biol..

[45] Puri P., Gosemann J.-H., Darlow J., Barton D.E. (2011). Genetics of vesicoureteral reflux. Nat. Rev. Urol..

[46] Lu M., Zhu K., Schulam P.G., Chai T.C. (2019). A non-enzymatic method for dissection of mouse bladder urothelial tissue. Nat. Protoc..

[47] Yu Z., Liao J., Chen Y., Zou C., Zhang H., Cheng J., Liu D., Li T., Zhang Q., Li J. (2019). Single-Cell Transcriptomic Map of the Human and Mouse Bladders. J. Am. Soc. Nephrol..

[48] Dupont S., Morsut L., Aragona M., Enzo E., Giulitti S., Cordenonsi M., Zanconato F., Le Digabel J., Forcato M., Bicciato S. (2011). Role of YAP/TAZ in mechanotransduction. Nature.

[49] Elosegui-Artola A., Andreu I., Beedle A.E.M., Lezamiz A., Uroz M., Kosmalska A.J., Oria R., Kechagia J.Z., Rico-Lastres P., Le Roux A.-L. (2017). Force Triggers YAP Nuclear Entry by Regulating Transport across Nuclear Pores. Cell.

[50] Massagué J., Sheppard D. (2023). TGF-β signaling in health and disease. Cell.

[51] Distler J.H.W., Györfi A.-H., Ramanujam M., Whitfield M.L., Königshoff M., Lafyatis R. (2019). Shared and distinct mechanisms of fibrosis. Nat. Rev. Rheumatol..

[52] Long Y., Niu Y., Liang K., Du Y. (2022). Mechanical communication in fibrosis progression. Trends Cell Biol..

[53] Leonardo-Sousa C., Barriga R., Florindo H.F., Acúrcio R.C., Guedes R.C. (2025). Structural insights and clinical advances in small-molecule inhibitors targeting TGF-β receptor I. Mol. Ther. Oncol..

[54] Meng X.-m., Nikolic-Paterson D.J., Lan H.Y. (2016). TGF-β: the master regulator of fibrosis. Nat. Rev. Nephrol..

[55] Eckstein M., Wirtz R.M., Gross-Weege M., Breyer J., Otto W., Stoehr R., Sikic D., Keck B., Eidt S., Burger M. (2018). mRNA-Expression of KRT5 and KRT20 Defines Distinct Prognostic Subgroups of Muscle-Invasive Urothelial Bladder Cancer Correlating with Histological Variants. Int. J. Mol. Sci..

[56] Ecke T.H., Kiani A., Schlomm T., Friedersdorff F., Rabien A., Jung K., Kilic E., Boström P., Tervahartiala M., Taimen P. (2020). Prognostic Role of Survivin and Macrophage Infiltration Quantified on Protein and mRNA Level in Molecular Subtypes Determined by RT-qPCR of KRT5, KRT20, and ERBB2 in Muscle-Invasive Bladder Cancer Treated by Adjuvant Chemotherapy. Int. J. Mol. Sci..

[57] Jütte H., Reike M., Wirtz R.M., Kriegmair M., Erben P., Tully K., Weyerer V., Eckstein M., Hartmann A., Eidt S. (2021). KRT20, KRT5, ESR1 and ERBB2 Expression Can Predict Pathologic Outcome in Patients Undergoing Neoadjuvant Chemotherapy and Radical Cystectomy for Muscle-Invasive Bladder Cancer. J. Personalized Med..

[58] Pei G., Yao Y., Yang Q., Wang M., Wang Y., Wu J., Wang P., Li Y., Zhu F., Yang J. (2019). Lymphangiogenesis in kidney and lymph node mediates renal inflammation and fibrosis. Sci. Adv..

[59] Baluk P., Naikawadi R.P., Kim S., Rodriguez F., Choi D., Hong Y.K., Wolters P.J., McDonald D.M. (2020). Lymphatic Proliferation Ameliorates Pulmonary Fibrosis after Lung Injury. Am. J. Pathol..

[60] Aldamanhori R., Chapple C.R. (2017). Underactive bladder, detrusor underactivity, definition, symptoms, epidemiology, etiopathogenesis, and risk factors. Curr. Opin. Urol..

[61] Hong X., Li S., Luo R., Yang M., Wu J., Chen S., Zhu S. (2024). Mechanisms of the TGF-β1/Smad3-signaling pathway in gender differences in alcoholic liver fibrosis. J. Physiol. Sci..

[62] Solopov P., Colunga Biancatelli R.M.L., Dimitropoulou C., Catravas J.D. (2021). Sex-Related Differences in Murine Models of Chemically Induced Pulmonary Fibrosis. Int. J. Mol. Sci..

[63] Cai C., Kilari S., Singh A.K., Zhao C., Simeon M.L., Misra A., Li Y., Misra S. (2020). Differences in Transforming Growth Factor-β1/BMP7 Signaling and Venous Fibrosis Contribute to Female Sex Differences in Arteriovenous Fistulas. J. Am. Heart Assoc..

[64] Ziller N., Kotolloshi R., Esmaeili M., Liebisch M., Mrowka R., Baniahmad A., Liehr T., Wolf G., Loeffler I. (2020). Sex Differences in Diabetes- and TGF-β1-Induced Renal Damage. Cells.

[65] Yu W., Huang X., Tian X., Zhang H., He L., Wang Y., Nie Y., Hu S., Lin Z., Zhou B. (2016). GATA4 regulates Fgf16 to promote heart repair after injury. Development.

[66] Guo C., Kaneko S., Sun Y., Huang Y., Vlodavsky I., Li X., Li Z.R., Li X. (2015). A mouse model of urofacial syndrome with dysfunctional urination. Hum. Mol. Genet..

[67] Otsubo A., Miyazato M., Oshiro T., Kimura R., Matsuo T., Miyata Y., Sakai H. (2021). Age-associated bladder and urethral coordination impairment and changes in urethral oxidative stress in rats. Life Sci..

[68] Li M., Chen X., Cao N., lv R., Gu B. (2022). Improvement of urethral dysfunction by 5-HT1A receptor agonist NLX-112 in diabetic rats. Neurourol. Urodyn..

[69] Xu Z., Zhang T., Chen H., Zhu Y., Lv Y., Zhang S., Chen J., Chen H., Yang L., Jiang W. (2023). High-throughput single nucleus total RNA sequencing of formalin-fixed paraffin-embedded tissues by snRandom-seq. Nat. Commun..

