# Supplementary material for: Keratin 20 deficiency phenocopies human UAB and drives bladder fibrosis via a mechanotransduction-TGF-β axis
Source: iScience. 2026 Apr 27;29(6):115905. doi: 10.1016/j.isci.2026.115905 (PMC13194166; doi:10.1016/j.isci.2026.115905)
Supplement: Document S1. Figures S1–S8 and Table S1 [file mmc1.pdf]

iScience, Volume *29*

Supplemental information

Keratin 20 deficiency phenocopies human UAB and drives bladder fibrosis via a mechanotransduction-TGF-β axis

Jun Jiang, Yongjia Bao, Tao Huang, Hanbo Zhang, Wensu Ma, Junwei He, Xianbin Duan, Chenxi Mo, Rui Guo, Jingjie Chen, Fang Yan, Jiehui Chen, Xing Liu, Yichen Huang, Fang Chen, Jiasheng Chen, Congcong Dong, and Chunming Guo

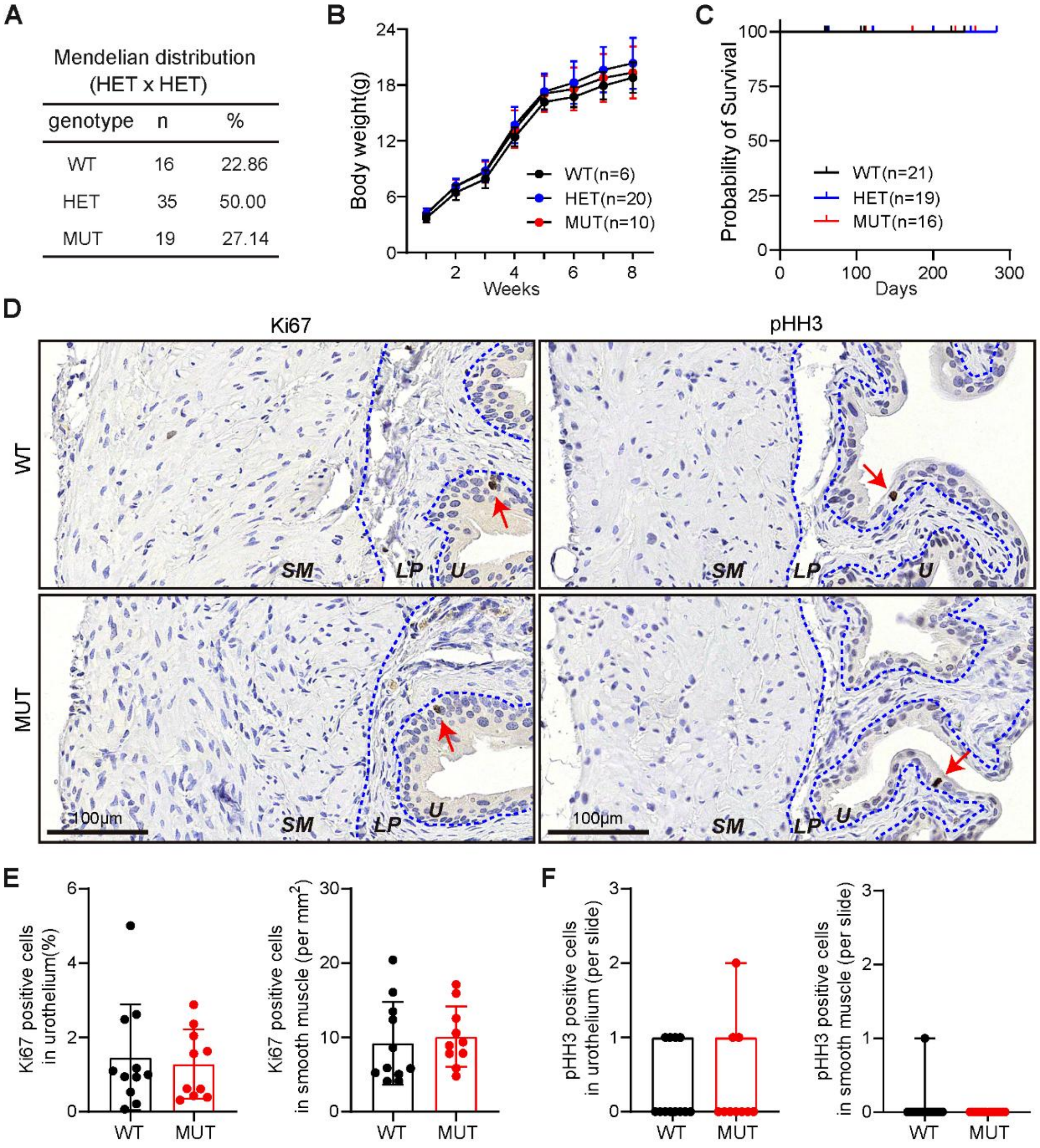

Figure S1. Krt20 deletion did not alter mendelian distribution, body weight and urothelial proliferation, related to Figure 1. (A) Mendelian ratio table for *Krt20* heterozygous mutant mating. **(B)** Body weight growth curves of *Krt20* mutant mice from 1 to 8 weeks. **(C)** Survival curves of *Krt20* mutant mice over 300 days. **(D)** Immunohistochemical staining of Ki67 and pHH3 in bladder tissue. Scale bar, 100μm. U, Urothelium; LP, lamina propria; SM, smooth muscle. **(E)** Statistical analysis of Ki67 positive cells in bladder urothelium and smooth muscle. **(F)** Statistical analysis of pHH3 positive cells in bladder urothelium and smooth muscle. Data are represented as mean ± SEM. Unpaired Student's T-test.

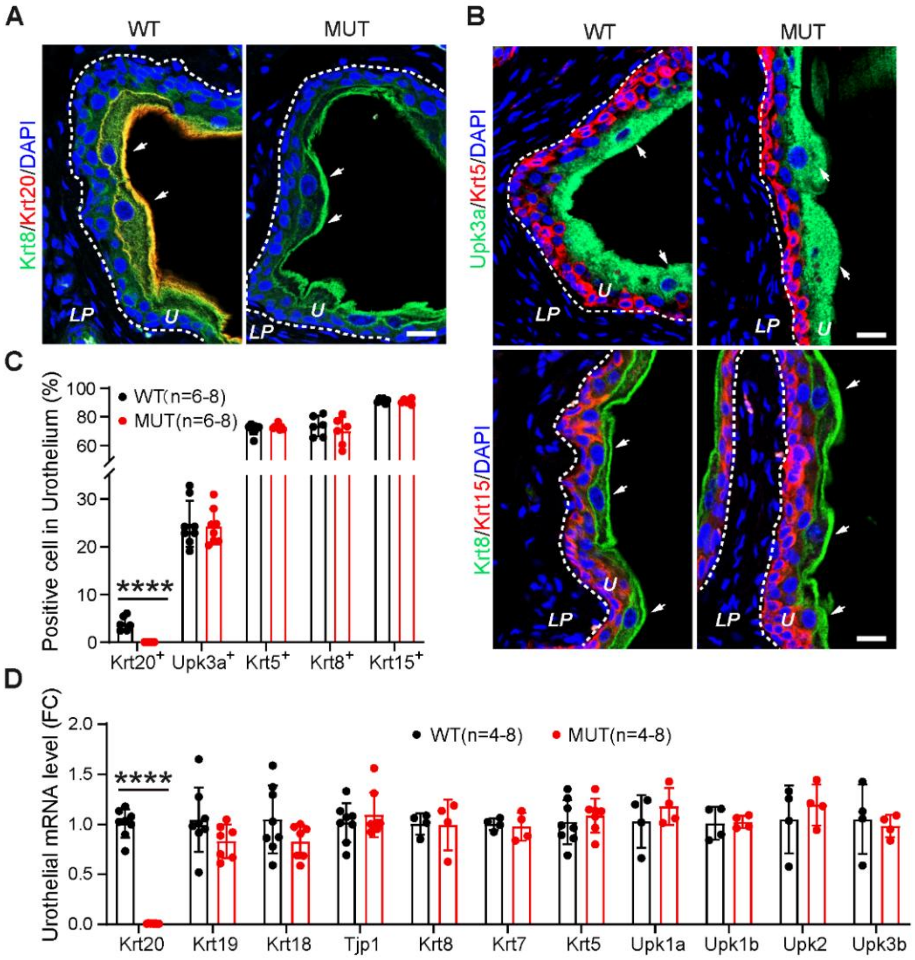

Figure S2. Krt20 deletion does not significantly alter the differentiation status of urothelial cells, related to Figure 1. (A-C) *Krt20* mutant mice had normal urothelial markers expression (A and B) and normal numbers of Krt5, Krt8, Krt15 and Upk3a-positive urothelial cells (C). (n=6-8 mice for each group). Scale bar, 20μm. Data are represented as mean ± SEM. Unpaired Student's T-test, ****, p<0.0001. **(D)** RT-qPCR analysis was used to detect the mRNA levels of urothelial markers in micro-dissected urothelial tissues. Data are represented as mean ± SEM. Unpaired Student's T-test, ****, p<0.0001.

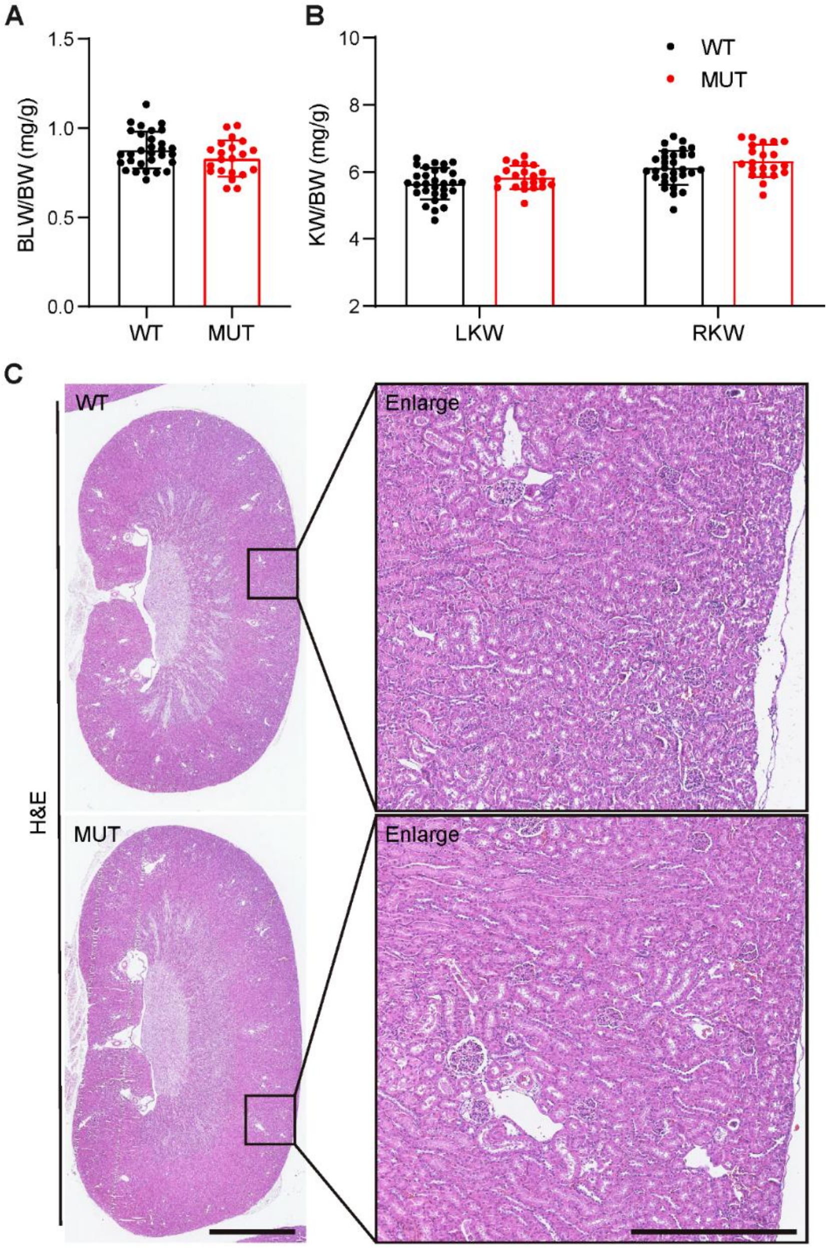

Figure S3. Krt20 did not alter bladder weight and kidney weight, mutant kidneys were barely seen abnormal if any, related to Figure 2. (A and B) Mouse bladder (A) and kidney weights (B) were normalized to body weight at 8 weeks of age. (WT, n=30, MUT, n=21). Data are represented as mean ± SEM. BLW, bladder weight; LKW, left kidney weight; RKW, right kidney weight; BW, body weight. **(C)** H&E pathological analysis of adult kidney from wild type and mutant mice. Scale bars, 2mm. Enlarge Scale bars, 500µm.

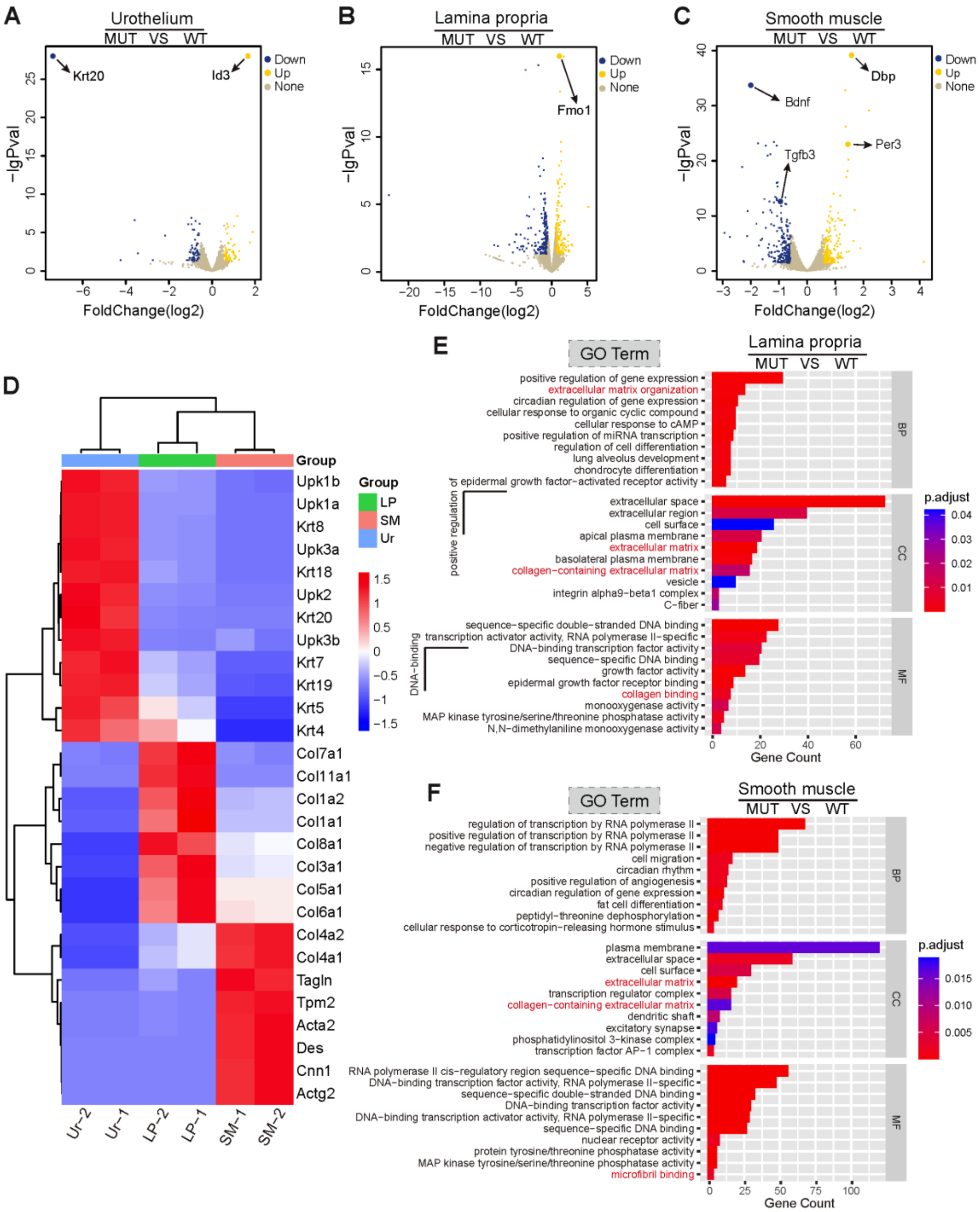

Figure S4. RNA-seq DEGs and GO term analysis for Micro-dissected bladder layers, related to Figure 3. (A-C) Volcano plot shows the differentially expressed genes (DEGs) in the urothelium (A), lamina propria (B), and smooth muscle (C) tissue layers of *Krt20* mutant mice. Group N=2, three mice were grouped in each. **(D)** Heat map shows the distribution of molecular markers in the urothelium, lamina propria, and smooth muscle tissue layers. **(E and F)** GO analysis showed the enrichment of GO terms in the lamina propria (E), and smooth muscle (F) tissue layers of *Krt20* mutant mice. Group N=2, three mice were grouped in each.

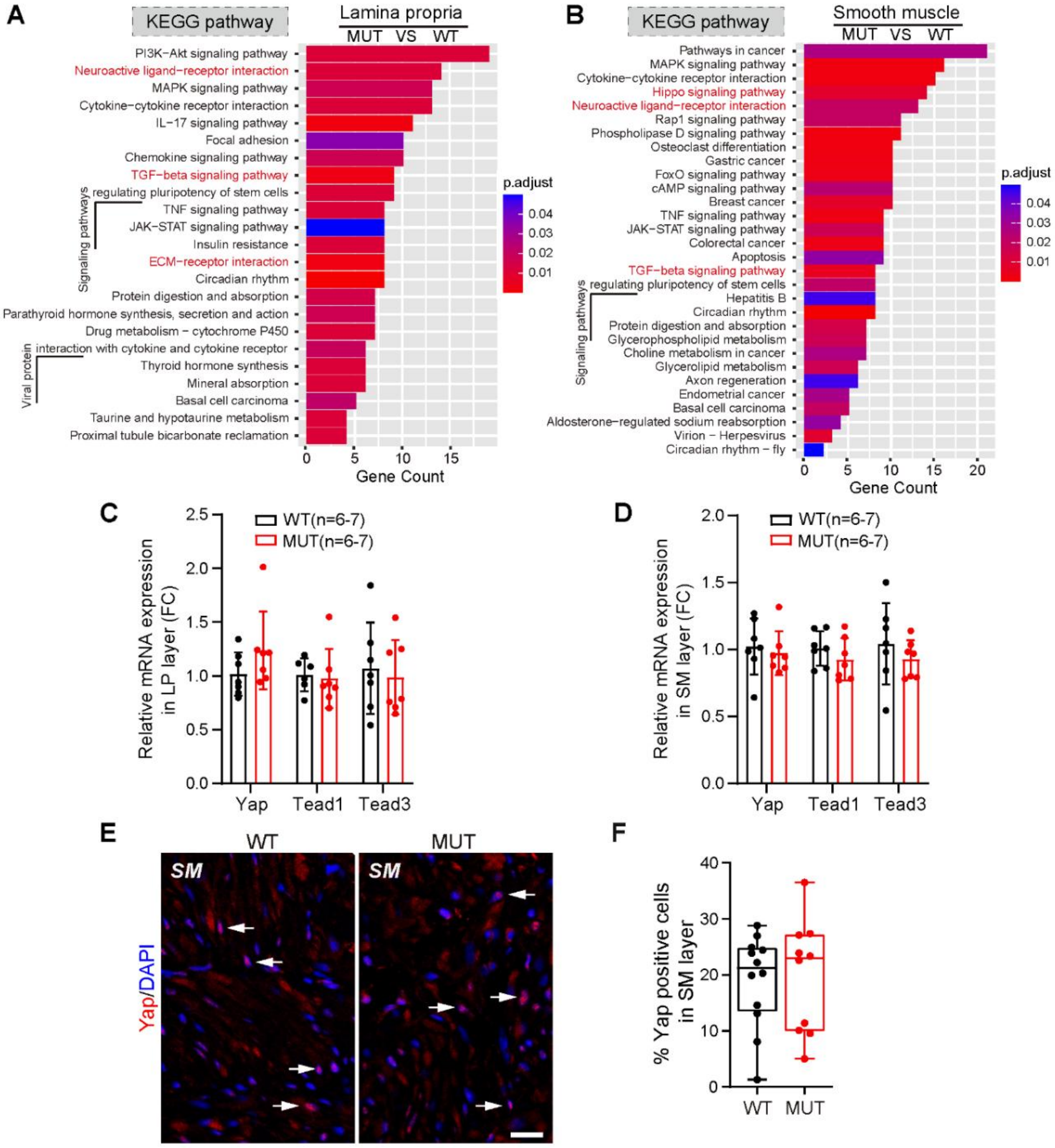

Figure S5. KEGG pathway analysis and Yap signaling pathway detection in the lamina propria and smooth muscle, related to Figure 3. (A and B) KEGG pathway analysis showed the enrichment of signaling pathway in the lamina propria (A) and smooth muscle (B) tissue layers of *Krt20* mutant mice. Group N=2, three mice were grouped in each. **(C and D)** RT-qPCR analysis was used to detect the expression of Yap signaling pathway-related genes in lamina propria (C) and smooth muscle (D) tissue layers. Data are represented as mean ± SEM. **(E and F)** Representative images (E) and statistical analysis of Yap nuclear translocation (F) in smooth muscle tissue layers. Scale bar, 20μm. SM, smooth muscle. Data are represented as mean ± SEM.

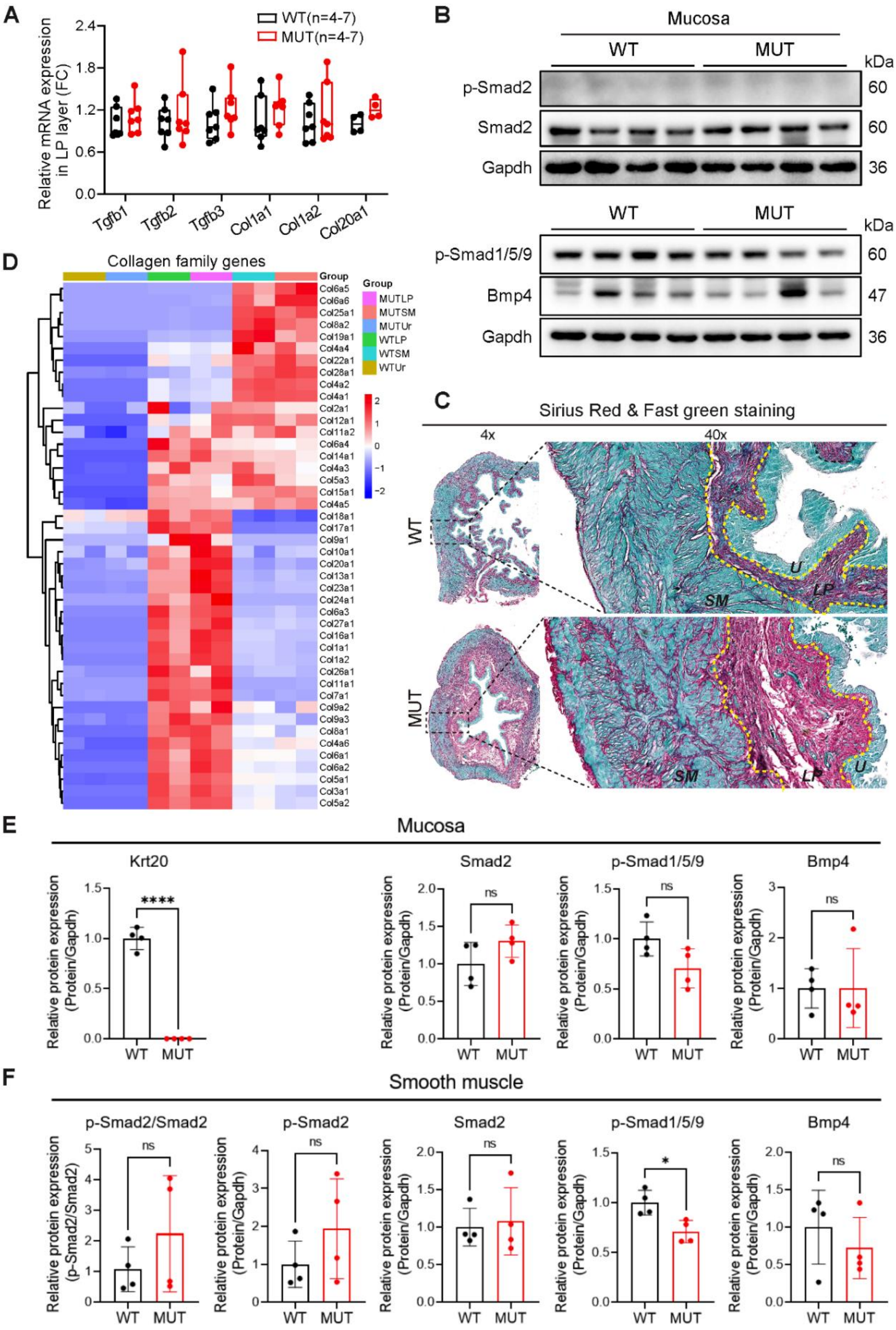

Figure S6. Krt20 deficient mice exhibited collagen family expression remodeling, related to Figure 4. (A) RT-qPCR analysis was used to further verify the TGF-β signaling pathway and downstream key genes in the lamina propria. Data are represented as mean ± SEM. **(B)** Protein expression and activation of TGF-β signal transducer in mucosa layer of mutant mice. **(C)**

Representative images of Sirius Red & fast green staining of bladder tissues in Krt20 mutant mice. U, Urothelium; LP, lamina propria; SM, smooth muscle. **(D)** Cluster analysis of collagen family's genes in the bladder tissue layer of Krt20 mutant mice. Ur/U, Urothelium; LP, lamina propria; SM, smooth muscle. Group N=2, three mice were grouped in each. **(E)** Relative quantitative statistics of TGF-β signal transducer expression and activation in mucosal layer (Protein/Gapdh). Data are represented as mean ± SEM. Unpaired Student's T-test, ****, p<0.0001. **(F)** Relative quantitative statistics of TGF-β signal transducer expression and activation in smooth muscle layer (Protein/Gapdh). Data are represented as mean ± SEM. Unpaired Student's T-test, *, p<0.05.

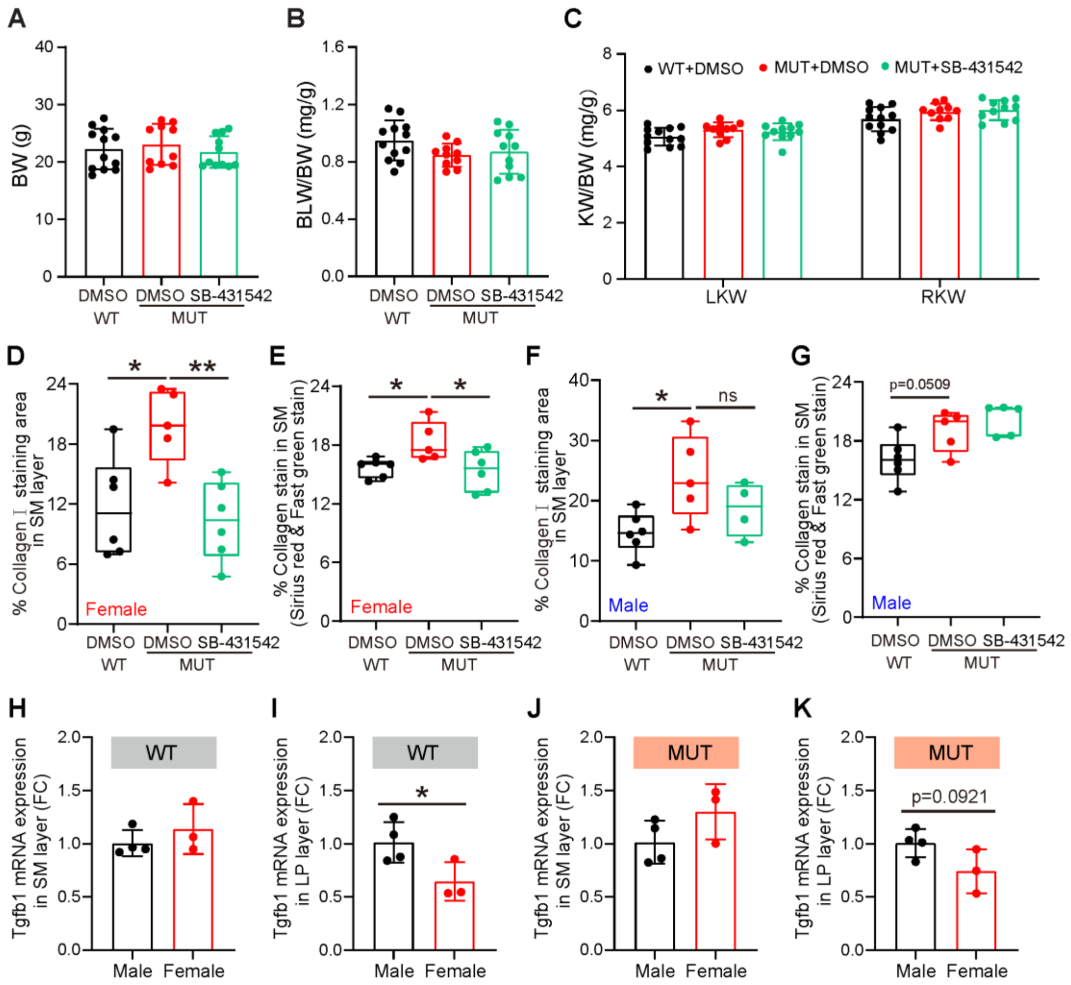

Figure S7. Alk4/5/7 inhibition alleviated *Krt20* mutant bladder fibrosis in a sexual dimorphic pattern, related to Figure 5. (A) Body weight analysis of mice treated with Alk4/5/7 inhibitor SB-431542 or DMSO. Each dot represents one mouse. **(B and C)** Bladder (B) and kidney (C) weights were normalized to body weight after treatment of mice with the Alk4/5/7 inhibitor SB-431542 or DMSO. Each dot represents one mouse. **(D and E)** Statistical analysis of type I collagen (D) and total collagen staining (E) after treatment with the Alk4/5/7 inhibitor SB-431542 or DMSO in female mice. Data are represented as mean ± SEM. Unpaired Student's T-test, *, p<0.05, **, p<0.01. **(F and G)** Statistical analysis of type I collagen (F) and total collagen staining (G) after treatment with the Alk4/5/7 inhibitor SB-431542 or DMSO in male mice. Data are represented as mean ± SEM. Unpaired Student's T-test, *, p<0.05. **(H and I)** Sex difference in *Tgfb1* expression in the bladder smooth muscle (H) and lamina

propria (I) layers of wild-type mice. Data are represented as mean ± SEM. Unpaired Student's T-test, *, $p<0.05$. **(J and K)** Sex difference in *Tgfb1* expression in the bladder smooth muscle (J) and lamina propria (K) layers of *Krt20* mutant mice.

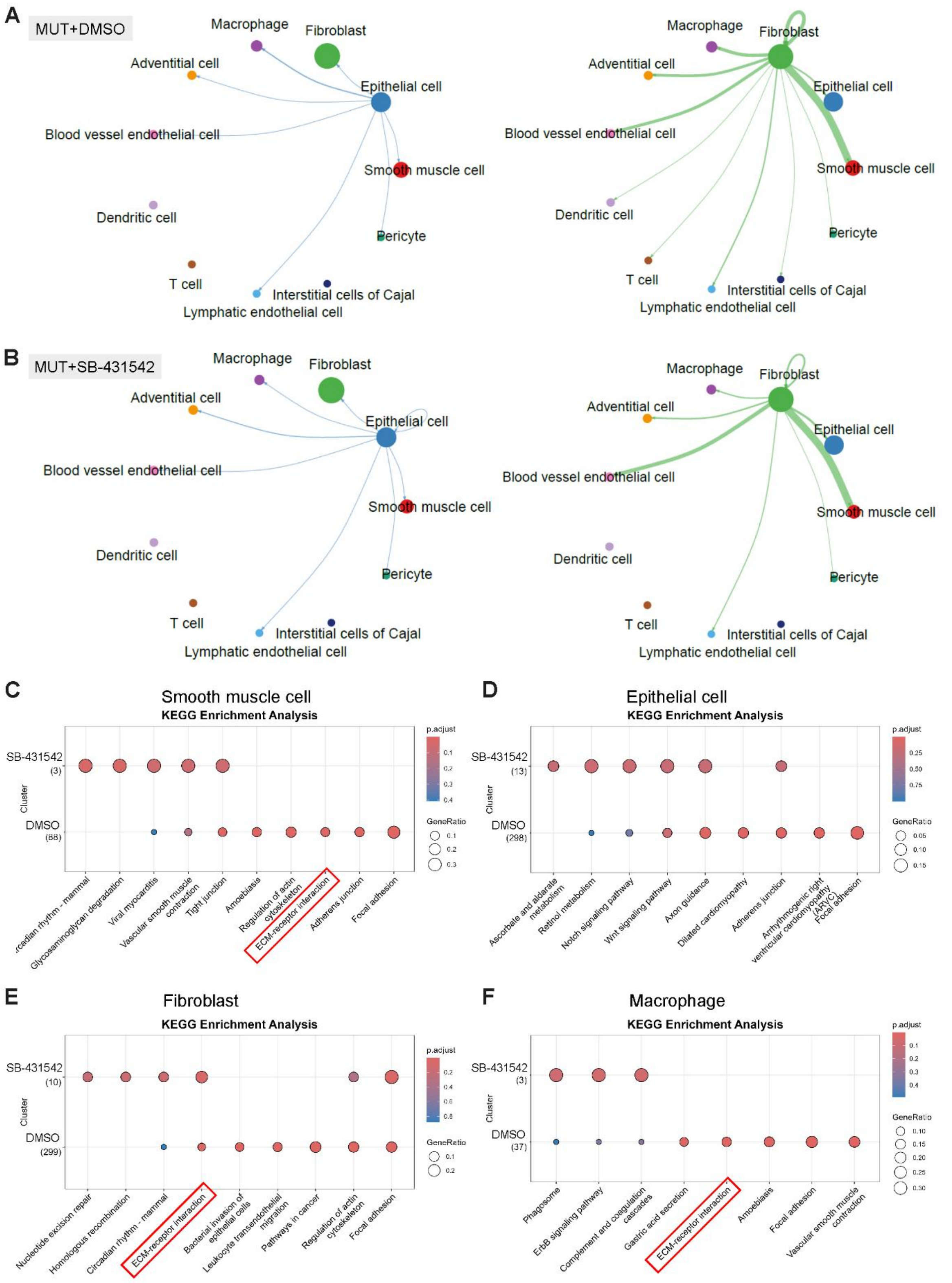

Figure S8. CellChat and KEGG enrichment analysis for the microenvironment perturbation by

Alk4/5/7 inhibition, related to Figure 6. (A and B) CellChat analysis of interactions among epithelial cells or fibroblasts towards to other cell types following SB-431542 intervention. **(C-F)** KEGG enrichment analysis for DEGs genes from MUT+DMSO group and MUT+SB-431542 group in smooth muscle cells (C), epithelial cells (D), fibroblasts (E) and macrophages (F).

Table S1. List of primers used in Genotyping and RT-qPCR

**Gene**	**Forward Primer (5'-3')**	**Reverse Primer (5'-3')**
**Genotyping experiments**		
WT	GAAAGGACACCCCTTCCTCG	GTTTCCCATTGCCCCCAAAC
Krt20 Mutant	GCTGTCTTGTTCGATGCCTG	CATCCTCAGGTTCAGCAGGG
**RT-qPCR experiments**		
Krt20 (mouse)	TGAGACAGAGATATGAAGTCCT	GCAGAGTTTGACTCTGTCTC
Krt19	GTTCAGTACGCATTGGGTCAG	GAGGACGAGGTCACGAAGC
Krt18	ACTGGTCTCAGCAGATTGAGG	CCGAGGCTGTTCTCCAAGTT
Tjp1	GCCGCTAAGAGCACAGCAA	GCCCTCCTTTTAACACATCAGA
Gapdh	GAAGGGCTCATGACCACAG	GATGCAGGGATGATGTTCTG
Krt5	TCTGCCATCACCCCATCTGT	CCTCCGCCAGAACTGTAGGA
Krt7	AGGAGATCAACCGACGCAC	GTCTCGTGAAGGGTCTTGAGG
Krt8	TCCATCAGGGTGACTCAGAAA	AAGGGGCTCAACAGGCTCT
Upk1a	CGTCGGACAGGCAACTTCAT	ACATCACAGGGAGGGTCCA
Upk1b	GGTGTAAACGGTCCGTCAGA	GGAGAACCCAAAAAGTCCAGC
Upk2	TGCCCCTGATCCTGATTCTG	CAAGGCAATTAACAGGCTTTCTG
Upk3b	AGACCTGATTGCCTACGTGC	GGTGTCCTTAGTTGAGACATGCT
Yap	TACTGATGCAGGTACTGCGG	TCAGGGATCTCAAAGGAGGAC
Tead1	AAGCTGAAGGTAACAAGCATGG	GCTGACGTAGGCTCAAACCC
Tead3	CAACCAGCACAATAGCGTCCA	CTGAAAGCTCTGCTCGATGTC
Tgfb1	CTGAACCAAGGAGACGGAATAC	GGGCTGATCCCGTTGATTT
Tgfb2	GTACCTTCGTGCCGTCTAATAA	GTGCCATCAATACCTGCAAATC
Tgfb3	CGCTACATAGGTGGCAAGAA	CAAGTTGGACTCTCTCCTCAAC
Col1a1	TGACCGATGGATTCCCGTTC	GCAGTGATAGGTGATGTTCTGG
Col1a2	GATGTTGAACTTGTTGCTGAGG	CAATGATTGTCTTGCCCCATTC
Col20a1	CCAAGGCTCCTACCAACACTT	GCGGCTACTTACTTGTCCCT
